# Reconstructing the reflectivity of liquid surfaces from grazing incidence X-ray off-specular scattering data

**DOI:** 10.1107/S1600576724002887

**Published:** 2024-05-17

**Authors:** Chen Shen, Honghu Zhang, Benjamin M. Ocko

**Affiliations:** a Deutsches Elektronen-Synchrotron DESY, Notkestrasse 85, 22607 Hamburg, Germany; b National Synchrotron Light Source II, Brookhaven National Laboratory, Upton, NY 11973, USA; Montanuniversität Leoben, Austria

**Keywords:** X-ray reflectivity, grazing incidence off-specular scattering, capillary waves, surface tension, diffuse scattering, surface-normal structure

## Abstract

The capillary wave model of a liquid surface predicts both the X-ray specular reflection and the diffuse scattering around it. A quantitative method is presented to obtain the X-ray reflectivity (XRR) from a liquid surface through the diffuse scattering data around the specular reflection measured using a grazing incidence X-ray off-specular scattering (GIXOS) geometry at a fixed horizontal offset angle with respect to the plane of incidence. With this approach the entire *Q_z_*-dependent reflectivity profile can be obtained at a single, fixed incident angle. This permits a much faster acquisition of the profile than with conventional reflectometry, where the incident angle must be scanned point by point to obtain a *Q_z_*-dependent profile. The XRR derived from the GIXOS-measured diffuse scattering, referred to in this paper as pseudo-reflectivity, provides a larger *Q_z_* range compared with the reflectivity measured by conventional reflectometry. Transforming the GIXOS-measured diffuse scattering profile to pseudo-XRR opens up the GIXOS method to widely available specular XRR analysis software tools. Here the GIXOS-derived pseudo-XRR is compared with the XRR measured by specular reflectometry from two simple vapor–liquid interfaces at different surface tension, and from a hexadecyltri­methyl­ammonium bromide monolayer on a water surface. For the simple liquids, excellent agreement (beyond 11 orders of magnitude in signal) is found between the two methods, supporting the approach of using GIXOS-measured diffuse scattering to derive reflectivities. Pseudo-XRR obtained at different horizontal offset angles with respect to the plane of incidence yields indistinguishable results, and this supports the robustness of the GIXOS-XRR approach. The pseudo-XRR method can be extended to soft thin films on a liquid surface, and criteria are established for the applicability of the approach.

## Abstract 2

Reflectivity from liquid surfaces can be accurately obtained from the diffuse scattering signal around the specular reflection measured by grazing incidence off-specular scattering experiments along with the capillary wave model of diffuse scattering. This method allows for a much faster reflectivity profile acquisition with an improved signal–noise ratio compared with conventional specular reflectometry.

## 1. Introduction

Liquid surfaces are important model systems for understanding the interactions near interfaces (Als-Nielsen & Pershan, 1983 ▸; Ocko *et al.*, 1997 ▸; Schwartz *et al.*, 1990 ▸; Haddad *et al.*, 2018 ▸; Pershan, 2011 ▸), and also serve as reaction platforms for electrochemistry (Sartori *et al.*, 2022 ▸), biophysics (Stefaniu *et al.*, 2014 ▸) or chemical engineering (Wang *et al.*, 2021 ▸). The use of reflectometry, X-ray (XRR) and neutron (NR), to study surface-induced layering and organic films on water is well established (Braslau *et al.*, 1988 ▸; Kjaer *et al.*, 1988 ▸; de Boer, 1994 ▸; Zhou & Chen, 1995 ▸; Pershan, 1994 ▸; Tolan, 1999*a* ▸; Daillant & Alba, 2000 ▸; Schlossman & Tikhonov, 2008 ▸), and it is the most reliable method for determining the surface-normal interfacial structure. To achieve the highest spatial resolution a large surface-normal scattering vector magnitude (*Q_z_*) is required. The limitation on the *Q_z_* range is typically when the reflected signal starts to be lower than about 20% of the background, and at these *Q_z_* values long counting times are required since the background-subtracted signal is much noisier than the total signal. This typically occurs when the reflectivity is of the order of 10^−9^, and for many systems this occurs at *Q_z_* > 0.5 Å^−1^. Long counting times limit the possibility of carrying out *operando* measure­ments and are often the reason for radiation damage on samples, especially at the highest *Q_z_* values where all attenuators have been removed and the sample footprint is the smallest. Moreover, performing liquid surface reflectometry requires accurate alignment and rotations of the deflecting crystal(s) in order to satisfy their Bragg condition(s) while the beam is deflected downwards over a wide range of incident angles (Als-Nielsen & Pershan, 1983 ▸; Schlossman *et al.*, 1997 ▸; Murphy *et al.*, 2014 ▸). The crystal-deflecting optics limit the availability of such liquid surface reflectometers, but also complicate the background shielding. Note that with NR the *Q_z_* range is typically about a factor of 2–2.5 less than with XRR (Campbell *et al.*, 2011 ▸). Despite the ability to enhance the contrast with NR through deuteration, for thin organic films XRR is often preferred over NR as it provides better spatial resolution.

Grazing incidence X-ray off-specular scattering (GIXOS) has been proposed as an alternative technique for measuring surface-normal interfacial structures on liquid surfaces (Wiegart *et al.*, 2005 ▸, 2009 ▸; Mora *et al.*, 2003 ▸; Dai *et al.*, 2011 ▸; Shen *et al.*, 2022 ▸). With this method the *Q_z_*-dependent diffuse scattering intensity is measured in a single shot at a fixed grazing incident angle. For liquid surfaces it is well established that the specular reflection (on or close to the specular axis) and diffuse scattering around the specular position (off-specular axis) are described by the same analytical scattering expressions (Tostmann *et al.*, 1999 ▸; Pershan, 2000 ▸; Shpyrko *et al.*, 2004 ▸), and the two signals cannot be separated. A combined analysis of the specular reflection and its diffuse scattering allows one to obtain both the root-mean-square (r.m.s.) roughness induced by the thermal excited capillary wave and the local, intrinsic interfacial structure on liquid surfaces (Sinha *et al.*, 1988 ▸; Shpyrko *et al.*, 2004 ▸; Daillant *et al.*, 2005 ▸; Vaknin, 2012 ▸). In pioneering GIXOS measurements on liquid surfaces, albeit over a limited *Q_z_* range, Dai *et al.* (2011 ▸) showed that with the incident angle less than the critical angle the diffuse scattering follows the expected form predicted by the capillary wave model. This suggests the possibility to analytically derive the reflectivity from GIXOS-measured diffuse scattering data, which would enable the use of fast and well established XRR analysis software tools and avoid the complicated and time-consuming analysis of the entire 2D diffuse scattering data. Here we refer to reflectivity that is derived from the GIXOS-measured diffuse scattering as ‘pseudo-reflectivity’, to distinguish it from the reflectometry-measured specular reflectivity where the exit scattering angle is always equal to the incident angle. Note that many efforts to describe the GIXOS from liquid surfaces are not complete or correct since the capillary wave contribution has been neglected (Wiegart *et al.*, 2005 ▸; Oliveira *et al.*, 2010 ▸; Pusterla *et al.*, 2022 ▸; Harvey *et al.*, 2023 ▸), and these efforts will be discussed further below.

Here we provide a simple expression to derive the pseudo-reflectivity from the diffuse scattering data measured with GIXOS. With this expression we find excellent agreement with specular reflectivity results up to *Q_z_* ∼ 1 Å^−1^. The calculation rests on the assumption that the expression for the diffuse scattering can be calculated precisely using the capillary wave model (CWM) and the measured sample must exhibit a homogeneous surface. Finally, we also show how the bending rigidity on liquid surfaces affects the calculation and its application to soft matter thin films on liquid surfaces.

## 2. Theory

Before presenting the relationship between the specular reflection from a liquid surface and its associated diffuse scattering, we review how the scattering intensity profile is related to the surface topology of the liquid surface. Readers who are mainly interested in the application of the pseudo-reflectivity method can jump directly to equations (8)–(10) at the end of this section.

The surface topology of a liquid surface is generated by the sum of thermally excited capillary waves. Their wavelengths extend from the atomic/molecular size to the gravitational cut-off, typically several millimetres. The amplitude of these capillary wave modes, which is the amplitude of the Fourier components of the height fluctuation 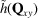 with the in-plane wavevector **Q** *_xy_*, can be related through the equipartition theorem to thermodynamic quantities (Bedeaux & Weeks, 1985 ▸). This provides the power spectral density (PSD), 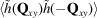 = 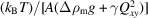 , where Δρ_m_, γ, *g*, *T*, *k*_B_ and *A* are, respectively, the mass density difference between the two sides of the interface, surface tension, gravitational constant, temperature, Boltzmann constant and surface area, and 〈…〉 denotes the ensemble average (Daillant & Alba, 2000 ▸; Bedeaux & Weeks, 1985 ▸; Tolan, 1999*b* ▸). A 2D Fourier transform of the PSD yields the real-space height–height correlation function of the instantaneous surface topology (Bedeaux & Weeks, 1985 ▸; Sanyal *et al.*, 1991 ▸; Tolan, 1999*b* ▸):

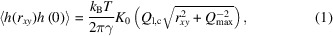

where *r_xy_* is the in-plane distance. 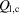 = 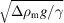 ∼ 10^−7^ Å^−1^ is the low-wavenumber cut-off above which the thermal capillary waves dominate and the long-wavelength waves cannot be excited. *K*_0_ is a hyperbolic Bessel function of the second kind of 0th order. For an average intermolecular distance *a*_m_, the shortest wavelength for capillary waves is 2*a*_m_ (neighboring molecules move in opposite directions) and accordingly *Q*_max_ = 2π/(2*a*_m_) = π/*a*_m_ is the upper-wavenumber cut-off for the capillary waves. The surface area term in the denominator of the PSD is canceled by an area term which appears in the numerator after performing a Fourier transform over the surface area.

**Figure 1:**
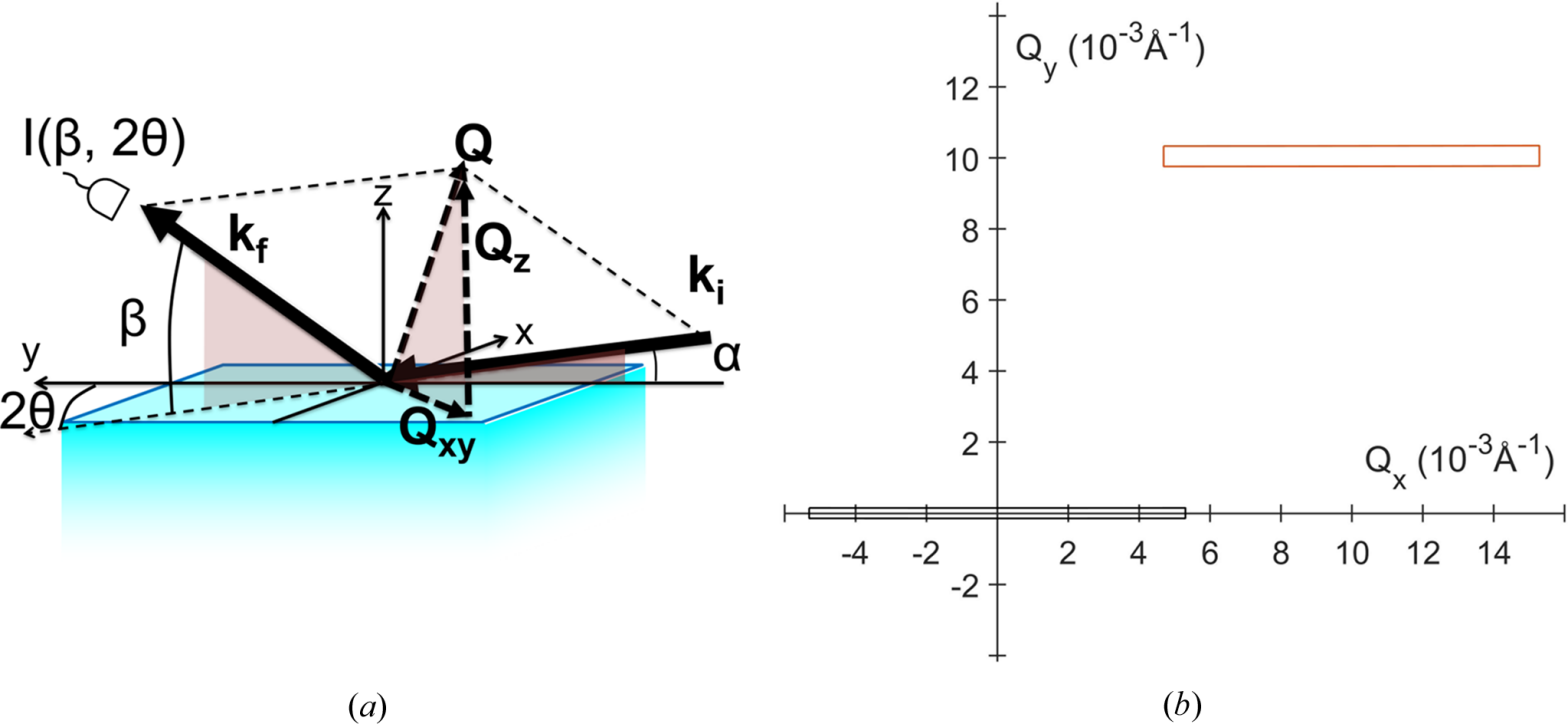
Schematics of (*a*) the geometry of an X-ray scattering experiment from a liquid surface (blue part) and (*b*) the resolution boxes in **Q** *_xy_* space that correspond to square areas on the detector in which the scattered photons are integrated (summed over), named as ‘area of integration’. (*a*) The sketch depicts the wavevectors **k**_i_ and **k**_f_ of the incident and the scattered beam, respectively, with a detector positioned at the angle (β, 2θ) to measure the intensity of the scattered beam. Plane *yz* is the plane of incidence. (*b*) The black resolution box represents the region of the intergral in **Q** *_xy_* space corresponding to a typical specular reflectometry measurement. It relates to the angular range of the integral in equation (4) used to calculate *R*. The red box represents the typical integral region for a GIXOS measurement and it relates to the integral angular range in equation (6) used to calculate 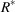 . [The example resolution boxes in (*b*) are calculated using a similar instrument setting to those used in our measurements: an incident energy of 15 keV, Δβ = Δ2θ = 0.08° set by an area of integration of 0.85 × 0.85 mm at 0.6 m from the sample. Both boxes are calculated for *Q_z_* = 0.4 Å^−1^: for the specular resolution (black) α = β = 1.5°, and for GIXOS resolution (red), α = 0.07°, β = 2.94°. 2θ for GIXOS is set to 0.0754°.] The **Q** *_xy_* center of the box is defined by (β, 2θ) of the center of the area of integration [see the calculation of (*Q_x_*, *Q_y_*)]: the box for the area of integration around a specular position (α = β, 2θ = 0) is centered at the origin of the **Q** *_xy_* space (black), while the center of the box around an off-specular position (α ≠ β, 2θ ≠ 0) is displaced from the origin (red).

The differential scattering cross section^1^ from the surface is computed from the Fourier transform of the height–height correlation function from real space into *Q* space (Braslau *et al.*, 1988 ▸; Pershan, 2000 ▸):

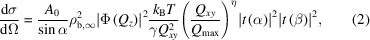

where 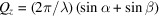 and the in-plane wavevector

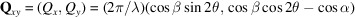

with the modulus 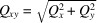 (Fig. 1 ▸). Ω is the solid angle at which the scattered flux is collected. *A*_0_ is the unit cross section area of the incident beam. λ is the X-ray wavelength. The incident angle is α, the vertical exit scattering angle is β and the horizontal (off-specular) exit scattering angle is 2θ (Fig. 1 ▸). The parameter 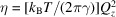 is a dimensionless exponent that scales with 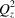 . The *z*-dependent scattering length density (SLD) profile is given by ρ_b_(*z*), where ρ_b,∞_ is the SLD of the bulk liquid. The SLD is related to the electron density ρ_e_ as ρ_b_(*z*) = ρ_e_(*z*)*r*_e_, where *r*_e_ = 2.82 × 10^−15^ m is the classical electron radius. The intrinsic surface-normal structure factor

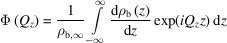

is the Fourier transform of the SLD gradient along the surface normal, normalized by ρ_b,∞_, and its squared modulus |Φ(*Q*_*z*_)|^2^ is used in the reflectivity calculation. The transmisson coefficients *t*(α) and *t*(β) are, respectively, the amplitudes of the evanescent wavefields induced by the incident and the scattering wave, and they are close to unity except near the critical incident/exit angle where they peak at about 2 (Feidenhans’l, 1989 ▸) (see Section S1 in the supporting information).

For both the specular reflection and the diffuse scattering around the specular reflection, the normalized intensity – the probability of an incident X-ray scattering into an angular opening ΔΩ – measured at an exit scattering angle (β, 2θ) is an integral of the differential cross section over ΔΩ (Braslau *et al.*, 1988 ▸). ΔΩ is defined by the vertical and horizontal angular openings Δβ and Δ2θ (FWHM) for measuring the scattered photons, as 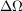 = 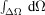 = 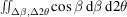 : 

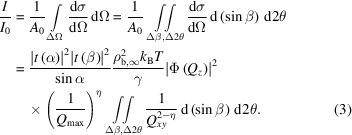




The angular openings Δβ and Δ2θ are the angular resolution of the measurements. The calculated differential 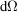 = 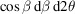 = 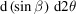 .

The measured specular reflectivity corresponds to the integral around the reflection condition (α = β) from 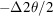 to 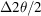 , and from 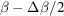 to 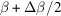 (Pershan, 2000 ▸):

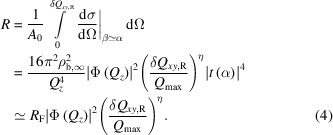

δ*Q*_*xy*,R_ is the in-plane *Q_xy_* resolution (HWHM) of the reflectivity measurement, which is defined by angular resolution Δβ and Δ2θ through the resolutions 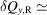 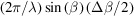 and 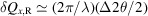 along *Q* *_y_* and *Q* *_x_*, respectively [Fig. 1 ▸(*b*)]. From a practical perspective, this is defined by a rectangular slit around the reflection condition in front of a point detector (Braslau *et al.*, 1988 ▸), or equivalently a rectangular area around the reflection condition on an area detector, within which the scattered photons are integrated [Fig. 1 ▸(*b*)]. For specular reflectivity, a fixed Δβ, determined by a constant vertical detector opening, is typically used over the entire β range. Hence the in-plane resolution δ*Q*_*xy*,R_ inceases with β and is *Q_z_* dependent. The *Q_z_* resolution δ*Q*_*z*,R_ is usually negligible for XRR since it is <0.1% of *Q_z_*, which is determined jointly by the energy dispersion and the angular divergence of the incident beam. Δβ contributes very little to the uncertainty of *Q_z_* since the integrand 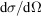 is very sharply peaked at β = α.

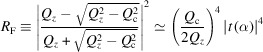

is the Fresnel reflectivity, *i.e.* the reflectivity of an ideal surface without roughness with a bulk SLD ρ_b,∞_, with *Q*_c_ = 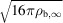 the critical wavevector (for a vapor–water interface at 293 K, *Q*_c_ = 0.02176 Å^−1^ for an X-ray energy of 15 keV). For α, β larger than several times the critical angle, |*t*(α)|^4^ is set to unity for the reason provided above. Under these conditions, the Fresnel reflectivity can be approximated as 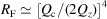 .

For simple liquid surfaces (no surface monolayer) it is convenient to assume that the intrinsic SLD profile ρ_b_(*z*) of the free liquid surface has an error function shape with an r.m.s. width of σ_0_, and hence its gradient dρ_b_(*z*)/d*z* has a Gaussian shape with the r.m.s. width of σ_0_ (Schwartz *et al.*, 1990 ▸). In this case, the phenomenological Gaussian r.m.s. roughness σ_R_ is provided by how the measured reflectivity falls off compared with the Fresnel reflectivity. This pheno­meno­logical roughness is given by

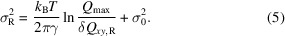

The first term is the summed contribution from all capillary wave modes, and the second term is the contribution from the r.m.s. width of the local, intrinsic SLD profile across the interface (Schwartz *et al.*, 1990 ▸; Pershan, 2000 ▸), which is related to the size of the atoms/molecules on the surface. This phenomenological roughness varies with the *Q_z_*-dependent in-plane resolution δ*Q*_*xy*,R_. It is common to calculate a single r.m.s. roughness value σ_R_ by using the δ*Q*_*xy*,R_ value at the largest *Q_z_* measured since the effect of σ_R_ is largest at the highest *Q_z_* values.

The diffuse scattering 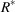 around the specular reflection has an analytical expression identical to that of *R* except for the range of integration: the range of integration of 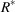 does not overlap the specular condition [Fig. 1 ▸(*b*)]. Using equation (3), 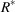 is given by

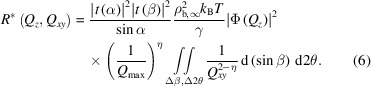




The in-plane *Q_xy_* resolution for 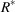 is also defined by Δβ and Δ2θ [Fig. 1 ▸(*b*)]. The out-of-plane *Q_z_* resolution δ*Q*_*z*_ for 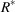 is determined differently from δ*Q*_*z*,R_ for specular reflectivity. As mentioned above, for specular reflectivity δ*Q*_*z*,R_ is effectively determined by the very narrow incident resolution and Δβ does not contribute to δ*Q*_*z*,R_. For diffuse scattering 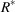 the integrand is slowly varying over the integrated range and, unlike the specular scattering, it does not exhibit a peak shape. Consequently the *Q_z_* resolution for the GIXOS case is given by 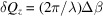 and this varies nearly linearly with Δβ. Hence, δ*Q*_*z*_ associated with 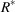 is larger than that associated with specular reflectivity. To increase the accepted 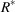 intensity it is straightforward to increase Δβ and Δ2θ. Note that this same principle does not apply for specular reflectivity. For thin surface films, this broader *Q_z_* resolution, compared with that of the specular reflectivity, has negligible effect on the structure-factor determination.

Rearranging the expression above provides the ratio *r*(*Q*_*z*_, *Q*_*xy*_) between the diffuse scattering 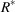 around the specular reflection and the specular reflectivity *R* at the same *Q_z_*:

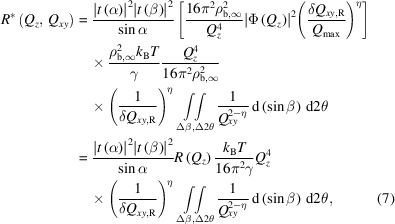




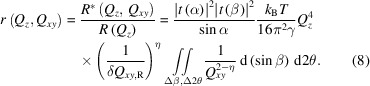




The first term is constant over most of the *Q_z_* range in the GIXOS geometry since the incident angle α is fixed (Dai *et al.*, 2011 ▸), and |*t*(β)|^2^ is close to unity except near the critical exit angle where it peaks at about 4 (Feidenhans’l, 1989 ▸). The second term is sample specific and depends on the temperature and surface tension. Except for the 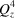 term, the *Q_z_* dependency arises from the final terms through η. Note that an in-plane resolution δ*Q*_*xy*,R_ of the pseudo-reflectivity must be specified in calculating *r*. As mentioned previously, for the conventional specular reflectivity case, the resolution δ*Q*_*xy*,R_ varies with *Q_z_* and consequently the r.m.s. roughness σ_R_ determined by conventional specular reflectivity depends on *Q_z_* [equation (5)]. The typical practice of using δ*Q*_*xy*,R_ from the highest *Q_z_* to calculate σ_R_ is only an approximation. However, the deviations of σ_R_ with this approximate form compared with the exact form using *Q_z_*-dependent δ*Q*_*xy*,R_ are relatively small since δ*Q*_*xy*,R_ appears in the logarithm of the σ_R_ calculation. Derivation of the pseudo-reflectivity allows the use of a single value of δ*Q*_*xy*,R_ for all *Q_z_* to provide a fixed value of σ_R_ that is independent of *Q_z_*. Note that to directly compare the pseudo-reflectivity *R*_pseudo_ (see below) with the measured specular reflectivity it is necessary to use the *Q_z_*-dependent form of δ*Q*_*xy*,R_ according to the reflectometer’s experimental configuration used in the calculation of *r*.

Δβ and Δ2θ are the angular integration ranges for the diffuse scattering measurement. If 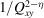 is slowly varying with 2θ over Δ2θ, it is appropriate to replace the integral by multiplying Δ2θ and Δβ with the averaged 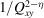 value between 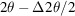 and 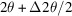 . Moreover, if we approximate |*t*(β)|^2^ as unity (see Section S1) the expression is further simplified to

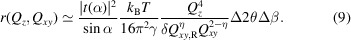




Equation (8) and its simplified version, equation (9), provide a quantitative means to derive the liquid surface specular reflectivity from the diffuse scattering around the specular reflection. This expression is accurate as long as the diffuse scattering can be fully described by the CWM. Here we refer to the reflectivity derived from the diffuse scattering around the specular reflection as pseudo-reflectivity *R*_pseudo_, to distinguish it from the measured reflectivity *R* by the conventional reflectometry method. It is calculated as

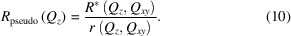




**Figure 2:**
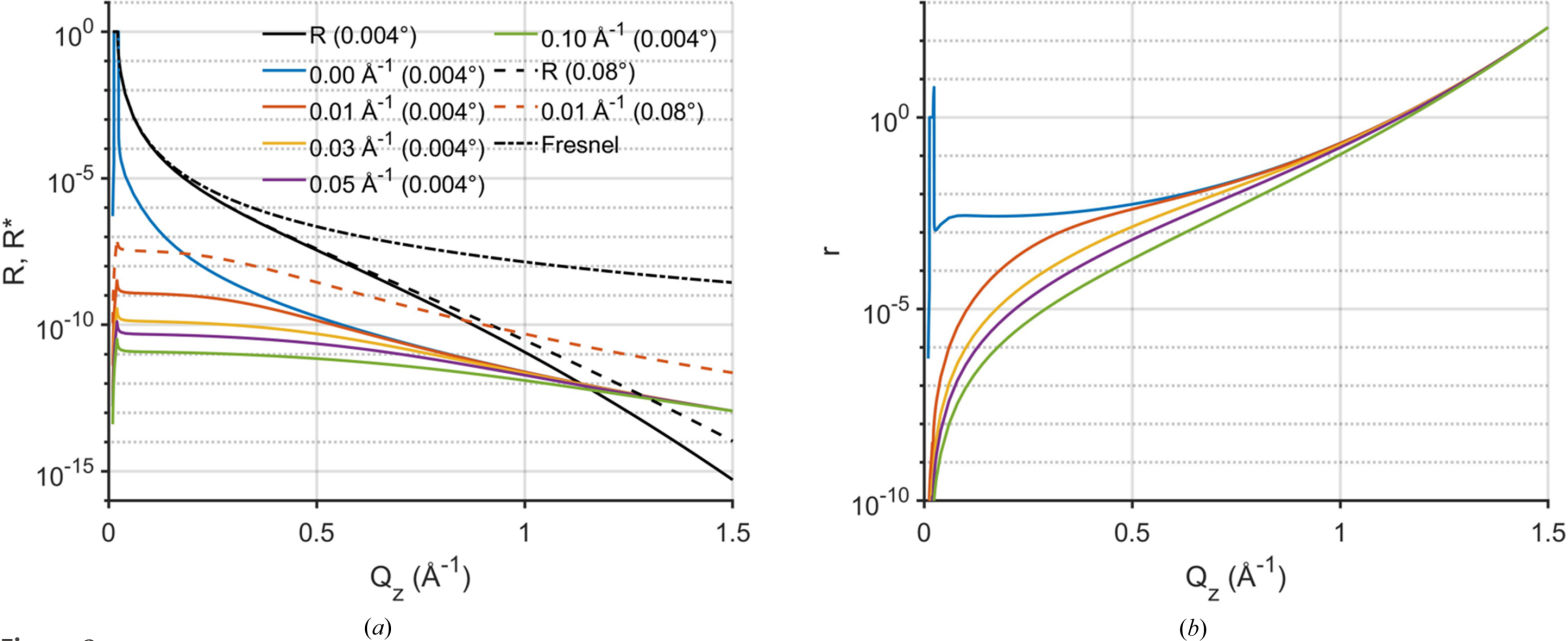
Theoretical courses of the grazing incidence diffuse scattering 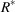 and of the specular reflectivity *R* from the water surface at 73 mN m^−1^ at 293 K (*a*), and their ratio *r* = 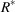 /*R* (*b*). The calculation assumes an incident energy of 15 keV, a vertical angular resolution Δβ = 0.08° and two different horizontal angular resolutions Δ2θ: 0.004° (solid lines) and 0.08° (dashed lines). The incident angle for 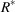 is set to 0.07°. 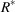 and *r* corresponding to different off-specular positions *Q*_*xy*_|_β=0_ are color coded [ 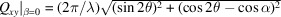 stands for *Q*_*xy*_ at β = 0]. Note that the blue lines are 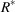 and *r* in the plane of incidence (*Q*_*xy*_|_β=0_ = 0 Å^−1^, *i.e.* 2θ = 0°). The legends provide the values of Δ2θ used (in parentheses), and the *Q*_*xy*_|_β=0_ value for 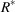 . In (*a*) the Fresnel reflectivity *R*_F_ (zero roughness, dash–dotted line) is shown as a reference. We use *Q*_*xy*_|_β=0_ rather than *Q*_*x*_|_β=0_ to define the off-specular position since at β= 0, *Q*_*xy*_ ≠ *Q*_*x*_. At this position, **Q** *_xy_* is not parallel to **Q** *_x_*. A small *Q_y_* component still persists [see the expression of *Q_x_* and *Q_y_* below equation (2)].

Two important features of the diffuse scattering around the specular reflection, shown by equations (6) and (8), provide the possibility to obtain the pseudo-reflectivity *R*_pseudo_ with a larger dynamic range than with conventional measured specular reflectivity *R*. One feature of 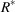 is the slower *Q_z_*-dependent decay compared with the 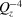 decay term of the specular reflectivity. This is demonstrated in Fig. 2 ▸ which shows that the *Q_z_* dependency of the diffuse scattering 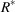 is relatively flat compared with that of *R*. Here, *R* and 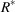 are computed with the same angular resolutions, Δβ = 0.08° and Δ2θ = 0.004°. The relative decay of these two [given by the *Q_z_* dependency of *r*, equation (9)] is 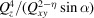 . Although 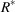 is relatively weak at small *Q_z_*, at sufficiently large *Q_z_* these two terms become comparable, with a value of 10^−12^ [*Q_z_* ∼ 1.1 Å^−1^, Fig. 2 ▸(*a*), black/colored solid lines]. As discussed above, the value of 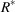 can be increased by increasing the angular resolutions Δβ and Δ2θ of the scattered photon measurement [equation (6)]. Fig. 2 ▸(*a*) shows a comparison between 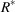 and *R* after a 20× broadening of the angular resolution (Δβ = 0.08°, Δ2θ = 0.08°, black/colored dashed lines). Over the whole *Q_z_* range, 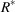 is increased by the same proportion (20×) as the broadening of the angular resolution, while *R* only increases slightly. Consequently, with a broader resolution 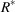 becomes comparable to *R* at much smaller wavevectors (*Q_z_* ∼ 0.8 Å^−1^, *R* ∼ 10^−10^). Above this value of *Q_z_*, the diffuse scattering 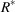 measured by GIXOS with a broader resolution is stronger than the specular reflectivity at the same *Q_z_*. Although 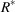 is small, about 10^−10^ to 10^−11^, it is readily measurable with grazing incidence experiments up to 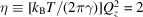 , *i.e.* the physical limit where the specular reflection from the liquid surface becomes absent (Pershan, 2000 ▸; Shpyrko *et al.*, 2004 ▸). In contrast, conventional specular reflectometry can only be reasonably measured for η < 1 since, at large *Q*_*z*_, the specular peak is very broad and the bulk scattering background is relatively strong (Shpyrko *et al.*, 2004 ▸). Hence, GIXOS makes it possible to measure the diffuse scattering and derive pseudo-reflectivities beyond the *Q_z_* range of conventional reflectometry, and this enables surface-normal structure analysis from liquid surfaces at improved real-space resolution. Additional examples are provided in the supporting information Section S2.

## 3. Instrument and experimental details

Experiments were conducted on two simple vapor–liquid interfaces at different surface tension [pure water and a mixture of water with 10 vol% ethanol (mass fraction 8%)] and the Gibbs layer of a 0.6 m*M* hexadecyltri­methyl­ammonium bromide (CTAB) solution in water. Pure water (resistivity > 18.2 MΩ cm at 25°C, total organic carbon < 2 p.p.b.) was obtained from the Purelab Ultra system (ELGA LabWater) or Millipore system (MilliporeSigma). Denatured ethanol (>99.8%, Carl Roth GmbH + Co. KG) was used as purchased. CTAB (>99.0%, Sigma–Aldrich), as purchased, was dissolved in pure water and stirred for 30 min at 40°C to give a 0.6 m*M* solution. All the glassware in contact with the liquid and the Langmuir trough plate was rinsed thoroughly with water, ethanol and water, three times each, before final filling with the liquid sample.

Experiments performed at the high-resolution diffraction beamline P08 at the PETRA III synchrotron (DESY, Hamburg, Germany) (Seeck *et al.*, 2012 ▸) utilize the Langmuir trough grazing incidence diffraction setup (Shen *et al.*, 2022 ▸). Experimental details are briefly summarized here while technical details of the setup are provided elsewhere (Shen *et al.*, 2022 ▸). The incident beam at 15 keV with a size of 0.25 × 0.07 mm (horizontal × vertical) was deflected downwards using a quartz mirror to an incident angle of 0.070° with respect to the horizontal plane. This corresponds to ∼85% of the critical angle of the air–water interface. The incident flux on the sample was ∼2 × 10^10^ photons s^−1^. To avoid air scattering, the X-ray beam was transported without windows in vaccum from the upstream optics to about 10 mm in front of the Langmuir trough, where it was terminated by a 25 µm-thick Kapton X-ray window. The trough setup (a modified G4, Kibron Inc., Finland) utilized a 350 mm-long Teflon trough, the temperature of which was controlled by circulating water beneath the Teflon trough. The enclosure was saturated with wet helium to reduce the background scattering (O_2_ mol% < 1%). To reduce the entrance window scattering (Shen *et al.*, 2022 ▸), a 0.3 mm-diameter pinhole in a 2 mm-thick tungsten sheet was placed within the trough enclosure, between the Kapton entrance window and the trough. A 0.5 mm-thick tungsten beamstop was installed within the trough enclosure, after the trough and before the Kapton exit window, in order to capture the specular reflected beam, and this minimizes small-angle scattering from the exit Kapton windows (for details of the beamstop, see Section S3). The front face of an Eiger2 X 1M detector (Dectris AG, Switzerland) was mounted 561 mm (*D*_det_) from the center of the Langmuir trough. No slit or collimation component was installed between the exit X-ray window of the trough enclosure and the detector. During the X-ray measurements the surface tension was monitored. The measurements of the CTAB/water solution surface, the ethanol solution and pure water were carried out with a circulating water temperature of, respectively, 292, 293 or 295 K (details above). A Pt100 resistance sensor (4-wire configuration) immersed in the subphase measured the same value as the set temperatures.

To measure the diffuse scattering around the specular reflection by GIXOS, the sample was illuminated for 140 s (∼3 × 10^12^ impinging photons). To measure the parasitic scattering from the enclosure chamber, the trough was lowered by 1 mm so that the beam was above the sample and directly illuminated the beamstop (same 140 s exposure). All other instrumental components remained at the same position.

Experiments performed at the Open Platform Liquids Scattering (OPLS) endstation of the beamline 12ID at the National Synchrotron Light Source II (NSLS-II, Brookhaven National Laboratory, USA) were carried out using a conventional single-crystal deflector where the sample height must be repositioned at each incident angle (Als-Nielsen & Pershan, 1983 ▸). This instrument, despite its higher background compared with the P08 instrument, offers both conventional specular XRR and GIXOS pseudo-reflectivity measurements, thus enabling a direct comparison of results from both methods on the same sample. At OPLS, measurements were carried out using 14.4 keV photons and a beam size of 200 × 7 µm (horizontal × vertical) at 292 K. In the XRR measurement, the specular reflection intensity was integrated over a square region of 1 × 1 mm on the Lambda 250k GaAs detector (X-Spectrum GmbH, Germany) mounted 1 m from the sample. This configuration corresponds to a rectangular angular opening with Δβ and Δ2θ of ±0.5 mrad. XRR background intensities were integrated over the same angular opening size, at 2θ = 1 mrad on both sides from the specular reflection position, and the two intensities were averaged. GIXOS measurements utilized an incident angle of 0.0723° and a PILATUS 100k detector (Dectris AG, Switzerland) at 0.6 m rotating about the *z* axis. The off-specular angle *Q*_*xy*_|_β=0_ = 0.04 Å^−1^ (see caption of Fig. 2 ▸) was set by the horizontal position of a 0.25 mm-wide post-sample slit. Scattering data were extracted from a 0.5 mm-wide (horizontal) region on the detector throughout its vertical length, and hence this region served the same purpose as the second slit in Fig. 1 of Fradin *et al.* (2000 ▸) and Dai *et al.* (2011 ▸). Within this region, the intensity was first summed through the horizontal direction and summed every 1 mm along the vertical direction to yield a 1D GIXOS profile along *Q*_*z*_ at a constant 2θ position with a resolution Δβ = 0.16° and Δ2θ = 0.08° (the value of Δβ corresponds to the binning size of 1 mm along the vertical direction, and the Δ2θ value corresponds to the 0.5 mm-wide region of integration, both for a 0.6 m detector–sample distance). The pros and cons of the configurations at P08 and at OPLS are discussed in Section S7.

To prepare the GIXOS data at both P08 and OPLS for integrals over solid angles, the raw detector images are first transformed into an intensity map *I*_raw_(β, 2θ) in angular space through geometric transformations that use the sample–detector distance *D*_det_, the pixel indices, the pixel size and the relative orientation of the incident beam with respect to the detector and the horizon orientations. Next, to enhance the signal–noise ratio, *I*_raw_(β, 2θ) from P08 is rebinned and grouped into uniformly spaced pixels in (β, 2θ) space with equal pixel sizes of Δβ = 0.08° and Δ2θ = 0.08° (see Section S5 which gives the intensity maps and an example region of rebinning). These approximately correspond to ten times the angular resolution of the pixel size of the detector. Details of the scattering angle calculation, rebinning, grouping and the geometrical correction used for these procedures are given in Section S4. Note that the rebinning into uniformly spaced pixels in (β, 2θ) space is not a necessary step for the analysis and is performed only for practical convenience. The binning and the angular resolution of the OPLS data were described in the previous paragraph.

Proper background subtraction is critical for extracting the contribution of the surface diffuse scattering 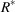 around the specular reflection from the GIXOS-measured signal. The instrument scattering data *I*_instr_(β, 2θ) are subtracted from the grouped data from the liquid surface *I*(β, 2θ) in order to remove the contributions from the window and the air scattering. In addition to this instrument contribution, one must also account for the scattering from the underlying bulk liquid (water, or the water/ethanol mixture) (Fradin *et al.*, 2000 ▸). Far from the plane of incidence, the contribution of the surface diffuse scattering originating from the interfacial thermal fluctuation is neglible and the bulk scattering is independent of the azimuthal angle. The large 2D scattering pattern from P08 allows one to obtain this *Q*-dependent, isotropic bulk liquid scattering by azimuthally averaging the wide-angle scattering – after the instrument scattering subtraction – at 2θ > 2.4° (*Q_xy_* > 0.3 Å^−1^) and β > 0.2° [azimuthal averaging was performed with *pyFAI* (Ashiotis *et al.*, 2015 ▸)]. This β range is larger than 2.5 times the critical angle such that the Yoneda peak (β = α_c_) is absent. The azimuthally averaged background has been phenomenologically modeled successfully using the form 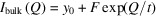 , up to *Q* = 1.3 Å^−1^, a *Q* range relevant for our GIXOS measurements, where *y*_0_, *F*, *t* are fitting parameters. The background-corrected data 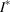 (β)|_2θ_ are then obtained by subtracting the bulk scattering intensity at the same *Q* to which (β, 2θ) corresponded:

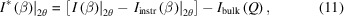

where

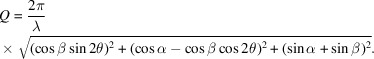

The limited *Q*_*xy*_ range of the OPLS-obtained GIXOS profile does not permit the aforementioned bulk scattering background method and instead a phenomenological approach is used. Details of background considerations relevant for performing a GIXOS experiment with the two configurations can be found in Section S7. Finally, normalizing 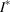 (β)|_2θ_ by the primary beam intensity yields the diffuse scattering 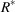 (β)|_2θ_ around the specular reflection.

For the two simple liquid surfaces, the pseudo-reflectivity is derived from the diffuse scattering data 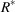 (β)|_2θ_ at different 2θ by equations (8)–(10) for a pseudo-reflectivity resolution δ*Q*_*xy*,R_ = 2 × 10^−4^ Å^−1^, using the chosen angular resolutions, Δ2θ = 0.08°, Δβ = 0.08°, of the diffuse scattering 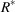 , and the measured surface tension and temperature values. The choice of δ*Q*_*xy*,R_ = 2 × 10^−4^ Å^−1^ is predicated on previous measurements (Schwartz *et al.*, 1990 ▸; Vaknin *et al.*, 2009 ▸).^2^ *Q_xy_* is computed for each β as described in Section 2. The CWM [equation (4)] has already been shown to correctly describe the specular reflectivity for several simple liquids (Schwartz *et al.*, 1990 ▸; Sanyal *et al.*, 1991 ▸; Ocko *et al.*, 1997 ▸; Shpyrko *et al.*, 2004 ▸; Vaknin *et al.*, 2009 ▸). To validate the pseudo-reflectivity approach on these two simple liquids, we compare our results with previous specular reflectivity findings (Schwartz *et al.*, 1990 ▸; Shpyrko *et al.*, 2004 ▸; Vaknin *et al.*, 2009 ▸). To derive the pseudo-reflectivities we set the intermolecular distance *a*_m_ to 3.1 Å for water (Schwartz *et al.*, 1990 ▸) and to 3.4 Å for the water/ethanol mixture (Daillant *et al.*, 2005 ▸). The structure factor (modulus) can be obtained according to equation (4) using the measured, normalized pseudo-reflectivity and a CWM term:

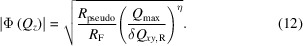




An error function is used as the model SLD profile of the simple liquid surfaces to fit the local intrinsic structure factor |Φ(*Q*_*z*_)| in order to obtain the intrinsic r.m.s. roughness σ_0_ of the interface. Note that the same expression applies for specular reflectivity by replacing *R*_pseudo_ by *R*.

In the case of the CTAB monolayer, the *Q*_*z*_-dependent δ*Q*_*xy*,R_ resolution at OPLS corresponding to an angular opening Δβ and Δ2θ of ±0.5 mrad is applied to the derivation of *R*_pseudo_ to allow a direct comparison with the specular XRR measured on the same sample. Moreover, one must also consider how the background is subtracted in the OPLS XRR measurement since the scattering background also includes a small diffuse scattering component (Section S8) (Pershan, 2000 ▸). The background was obtained by measurements of the off-specular signal at 1 mrad in 2θ. Hence the background-subtracted measured XRR is not precisely *R* given in equation (4) but *R*′,

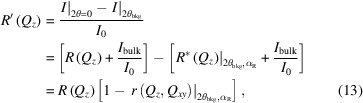

and it is often more conventient to express this as

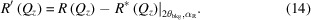




Here *R*′ and 2θ_bkg_ (1 mrad) are, respectively, the conventionally measured specular reflectivity and the off-specular angle of the background measurement, where 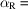 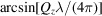 is the XRR incident angle for *Q*_*z*_. *I*|_2θ=0_ and 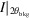 are the intensities at the specular and the off-axis (2θ_bkg_) positions, both measured with the angular opening Δβ and Δ2θ of ±0.5 mrad. *I*_bulk_ is the contribution of the bulk scattering background, assumed to be the same at the specular and slight off-axis positions. 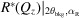 and 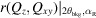 are the diffuse scattering contribution at the 2θ_bkg_ off-specular angle from the specular reflection position (and hence with the incident angle α_R_) and its ratio to the specular reflectivity, respectively. *R*′ is always smaller than *R* calculated by equation (4) since the signal measured at 2θ_bkg_ contains remnants of the surface diffuse scattering due to the 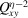 tails (Shpyrko *et al.*, 2004 ▸). This deviation is negligible at small *Q*_*z*_ and becomes significant when the diffuse scattering around the specular reflection approaches the order of the reflection at large *Q*_*z*_. For comparison with *R*′, a corresponding 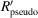 that also subtracts an off-axis diffuse scattering must be used,

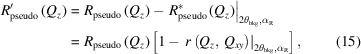

where 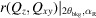 is computed with the configuration of the OPLS XRR measurement: Δβ = ±0.5 mrad, Δ2θ = ±0.5 mrad, 2θ_bkg_ = 1 mrad and 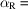 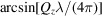 .

## 4. Simple liquid surfaces

**Figure 3:**
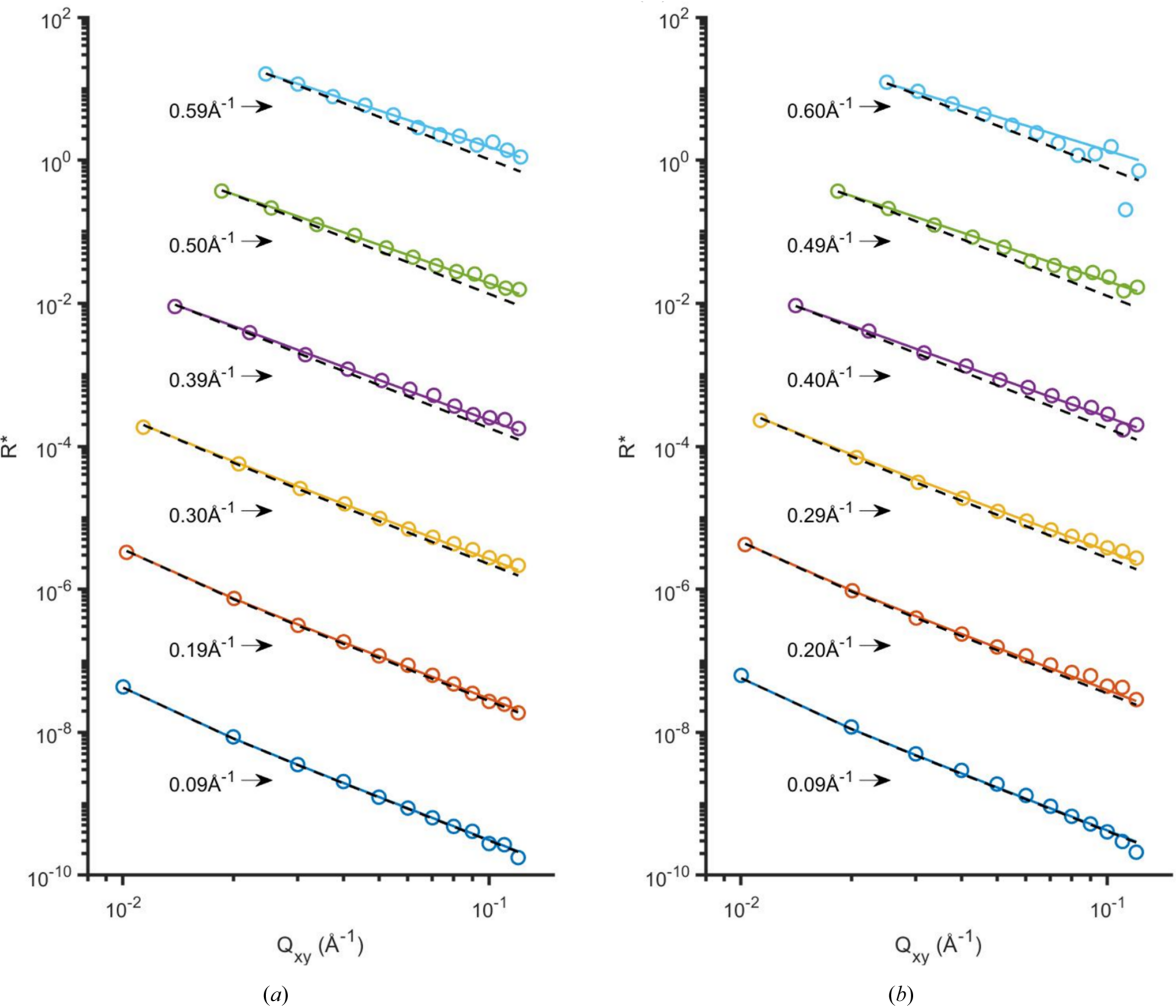
*Q_xy_* dependency of 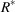 from the water surface (*a*) and from the surface of the mixture of water with 10 vol% ethanol (*b*). The surface tensions are 73 and 53 mN m^−1^ (Vazquez *et al.*, 1995 ▸), at 295 and 293 K, respectively. Data (circles) at different *Q_z_* are color coded and offset by a factor of 10 from each other, while the corresponding *Q_z_* values are given on the left. Solid lines are the theoretical 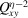 drop given by the tension and temperature at each *Q_z_* according to equation (6). The black dashed lines show simulated curves for 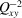 . These η = 0 curves, compared with the data, highlight that η must be non-zero to describe the curves for the largest *Q_z_* values.

Before comparing our results from simple liquid surfaces with literature reflectivity findings on the same systems, we first compare the diffuse scattering results with the CWM to verify that the CWM is applicable to our results. In Fig. 3 ▸ we show the *Q_xy_* dependency of the diffuse scattering 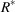 around the specular reflection, measured by GIXOS, for the water surface and for the surface of a mixture of water with 10 vol% ethanol (mass fraction 8%). They both show the expected 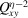 dependency of 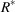 . The open circles are the measured 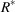 versus *Q_xy_* profiles at six different values of *Q_z_* ranging from 0.09 to 0.60 Å^−1^, and the solid lines display the theoretical 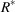 profiles predicted by CWM. Here η depends on the measured surface tension, temperature and *Q_z_*, and ranges from a value 0.009 at the smallest *Q_z_* to 0.320 at the largest *Q_z_* for water, and from 0.012 to 0.427 for the mixture. For all *Q_z_*, and for both samples, the results are in very good agreement with the theoretical 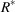 [lines, calculated from equation (6)] based on the CWM (Pershan, 2000 ▸). Note that the 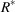 data were directly obtained by normalization to the measured primary beam intensity *I*_0_ as defined by equations (3) and (6), and no further renormalization has been performed (see Section 3). Further consideration of the normalization of 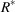 can be found in Section S9.

**Figure 4:**
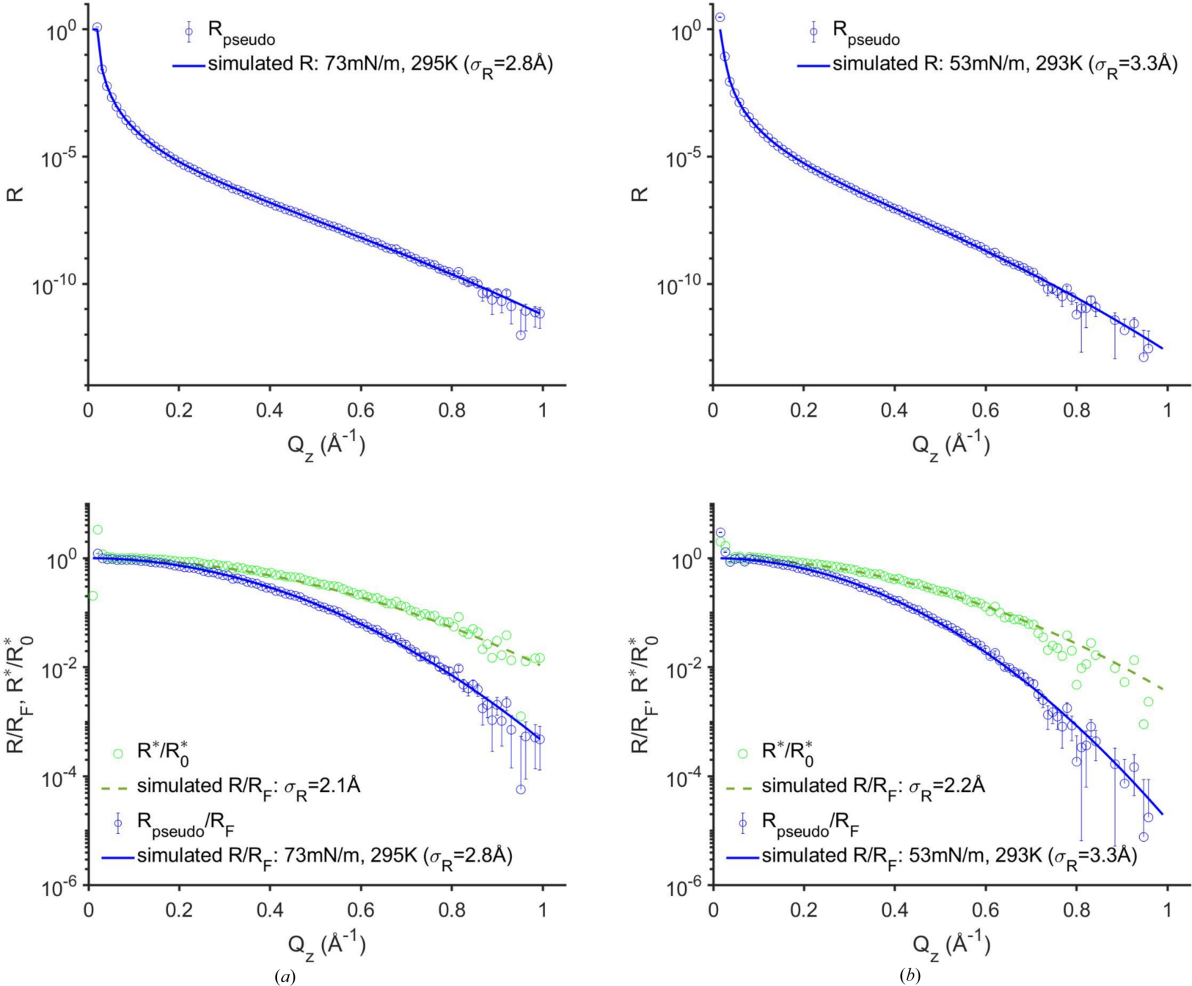
The diffuse scattering data measured by GIXOS at *Q*_*xy*_|_β=0_ = 0.03 Å^−1^ (see Fig. 2 caption) and the pseudo-reflectivity data derived from it, from the water surface (*a*) and from the surface of the mixture of water with 10 vol% ethanol (*b*). The blue circles (with error bars) show *R*_pseudo_ = 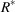 /*r* (top) and *R*_pseudo_/*R*_F_ (bottom) along with the simulated *R*/*R*_F_ predicted by the CWM (solid blue lines, see text). The green circles correspond to the normalized diffuse scattering, 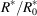 , where 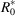 = 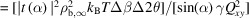 is the *Q*_*z*_-independent pre-factor of 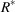 [equation (6)]. The normalized diffuse scattering follows *R*/*R*_F_ with a smaller r.m.s. roughness predicted by the CWM (green dashed line, see text).

The pseudo-reflectivity, *R*_pseudo_ = 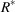 /*r*, derived from the GIXOS-measured diffuse scattering around the specular reflection from the two surfaces investigated is shown in Fig. 4 ▸. At the smallest *Q_z_* below *Q*_c_, *R*_pseudo_ is close to unity, as it is for specular reflectivity. At the largest *Q_z_* values (∼1 Å^−1^), the pseudo-reflectivity has fallen by about 11 orders of magnitude, about a factor of 10 larger than that which can be obtained with specular XRR. Over the entire *Q_z_* range, the pseudo-reflectivity (δ*Q*_*xy*,R_ = 2 × 10^−4^ Å^−1^) agrees very well with the theoretical specular XRR curve predicted by the CWM [equation (4)]. Here, the specular XRR consists of the product of three terms: (i) *R*_F_ using ρ_b,∞_ of the bulk, (ii) the CWM term 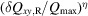 , obtained using the measured surface tension and temperature and the pseudo-reflectivity resolution δ*Q*_*xy*,R_ = 2 × 10^−4^ Å^−1^, and (iii) the instrinsic structure-factor term which is given by a Gaussian r.m.s. roughness whose value is obtained via a fit. For our measurements, the fitted intrinsic roughness for water is σ_0_ = 0.4 ± 0.1 Å and for the water mixed with 10% ethanol the fitted σ_0_ = 0.9 ± 0.1 Å. The water result is in excellent agreement with σ_0_ = 0.5 Å obtained by Shpyrko using specular XRR (Shpyrko *et al.*, 2004 ▸) (although Shpyrko *et al.* do not explicitly give the value of the intrinsic roughness, the negative deviation in their Fig. 4 corresponds to a σ_0_ of 0.5 Å), an experiment that properly accounted for the diffuse scattering (see Section S8). Our measured intrinsic roughness is close to the atomic radius of oxygen, 0.6 Å (Slater, 1964 ▸), which is by far the dominant scattering element of water. Our results are also in reasonable agreement with the GIXOS-measured diffuse scattering results of Dai *et al.* (2011 ▸) who obtained σ_0_ = 0 Å, albeit with a much larger error bar given the reduced range of the measurements and the absence of the bulk contribution subtraction. Our results would also be in reasonable agreement with those of Schwartz (0.85 Å) or Vaknin (0.6 Å),^3^ if they had used the same *Q*_max_ = π/*a*_m_ as used here (Schwartz *et al.*, 1990 ▸; Vaknin *et al.*, 2009 ▸). Measurements from other studies obtained larger values of σ_0_ which may originate from the cleanness of the water surface and/or the lower XRR values at large *Q_z_* that originate from an overestimation of the background (Braslau *et al.*, 1985 ▸, 1988 ▸; Pershan, 2016 ▸; Murphy *et al.*, 2014 ▸). This is because their background included remnants of the surface diffuse scattering signal (see Section S8). Compared with the pioneering GIXOS studies of Dai *et al.* (2011 ▸), which extend in *Q_z_* to 0.5 Å^−1^, albeit with error bars larger than that of the specular XRR, our results double the *Q_z_* range (1 Å^−1^) and have a smaller uncertainty.

**Figure 5:**
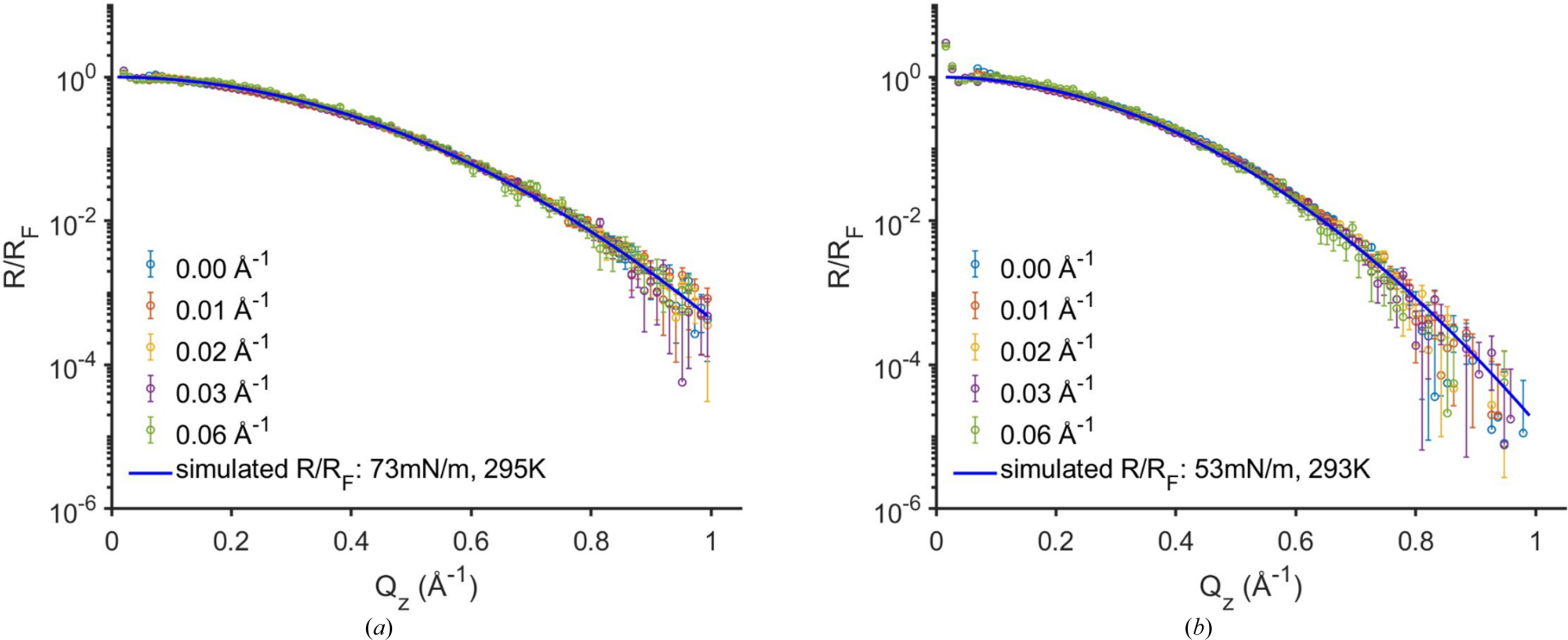
Pseudo-reflectivity results (normalized by *R*_F_) derived from GIXOS-measured diffuse scattering data at different off-specular positions (color coded), from the water surface (*a*) and from the surface of the mixture of water with 10 vol% ethanol (*b*). The off-specular positions *Q*_*xy*_|_β = 0_ are given in the legends (see Fig. 2 caption). Note that blue circles are derived from the data in the plane of incidence (*Q*_*xy*_|_β=0_ = 0 Å^−1^, *i.e.* 2θ = 0°).

Typically, the analysis of XRR from simple liquid surfaces involves fitting the entire deviation of *R*/*R*_F_ from unity by using a single phenomenological Gaussian r.m.s. roughness value, σ_R_ [equation (5)]. For the water and water/ethanol mixture, this yields σ_R_ of 2.8 ± 0.1 and 3.3 ± 0.1 Å, respectively, where we have used an in-plane reflectivity resolution, 2 × 10^−4^ Å^−1^, that is similar to the XRR resolutions utilized by Schwartz *et al.* (1990 ▸) and Vaknin *et al.* (2009 ▸) to calculate *R*_pseudo_ from 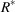 . These phenomenological Gaussian roughness values are consistent with the literature values (Schwartz *et al.*, 1990 ▸; Vaknin *et al.*, 2009 ▸). Directly fitting the deviation of 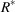 from unity rather than using ( 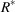 /*r*)/*R*_F_ yields an underestimated r.m.s. roughness (σ_R_ ≃ 2 Å, Fig. 4 ▸), *i.e.* the interface would incorrectly appear to be sharper. Note that in earlier GIXOS publications the analyses had used 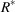 (equivalent to setting *r* ∝ 1/*R*_F_) and those analyses did not consider the diffuse scattering effects of the CWM (Oliveira *et al.*, 2010 ▸; Pusterla *et al.*, 2022 ▸; Harvey *et al.*, 2023 ▸). Despite the excellent fits and a reasonable qualitative estimation of the SLD profiles ρ_b_(*z*), the fitted values of σ_R_ were unphysically small, similar to the water example discussed above. According to our expressions, the value of ( 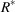 /*r*)/*R*_F_ should be independent of the off-specular position. As demonstrated in Fig. 5 ▸ for five off-specular positions (*Q*_*xy*_|_β=0_: 0, 0.01, 0.02, 0.03 and 0.06 Å^−1^) (see Fig. 2 caption), the ( 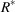 /*r*)/*R*_F_ curves are independent of the position. [Note that that 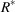 /*R*_F_ profiles at different off-specular positions *Q*_*xy*_|_β=0_ do not overlap (Section S6, Fig. S5).] This invariance demonstrates that the derivation of *R*_pseudo_ = 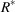 /*r* correctly accounts for the *Q_xy_* dependence of the CWM. Although the GIXOS-XRR method can be applied to any off-specular position, smaller positions yield more precision as they have better counting statistics and signal-to-background ratios.

## 5. CTAB Gibbs monolayer

The calculation of *R*_pseudo_ = 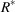 /*r* through the measurement of 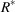 and the calculated *r* should also be applicable to soft matter thin films on a liquid surface as long as the film bending modulus κ_c_ is sufficiently small. In this case the surface topology is still dominated by the capillary wave of the subphase liquid, and the effect of the film rigidity is negligible. To see whether equations (8) and (9) are applicable, one must evaluate the expression of the PSD 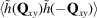 = 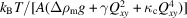 (Tolan, 1999*b* ▸) to check the contribution of the 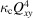 term. When either the bending rigidity or *Q_xy_* is sufficiently small such that 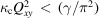 , the effects of bending rigidity can be ignored in the analysis, and equations (8) and (9) are a good approximation for calculating *r* (results to be published). One must consider that *Q_xy_* increases with β (Section S10) since *Q_y_* increases with β (see Fig. 2 caption). Here we calculate the *Q_xy_* value at the highest *Q_z_* (largest β) of the measurements. Next, this value is used to calculate the maximum 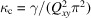 where equations (8) and (9) remain a good approximation. On the basis of the inequality above, for a maximum *Q_xy_* of 0.05 Å^−1^, and for a surface tension of 45 mN m^−1^ at a temperature of 292 K, equations (8) and (9) are applicable for a film with κ_c_ < 5*k*_B_ *T*.

**Figure 6:**
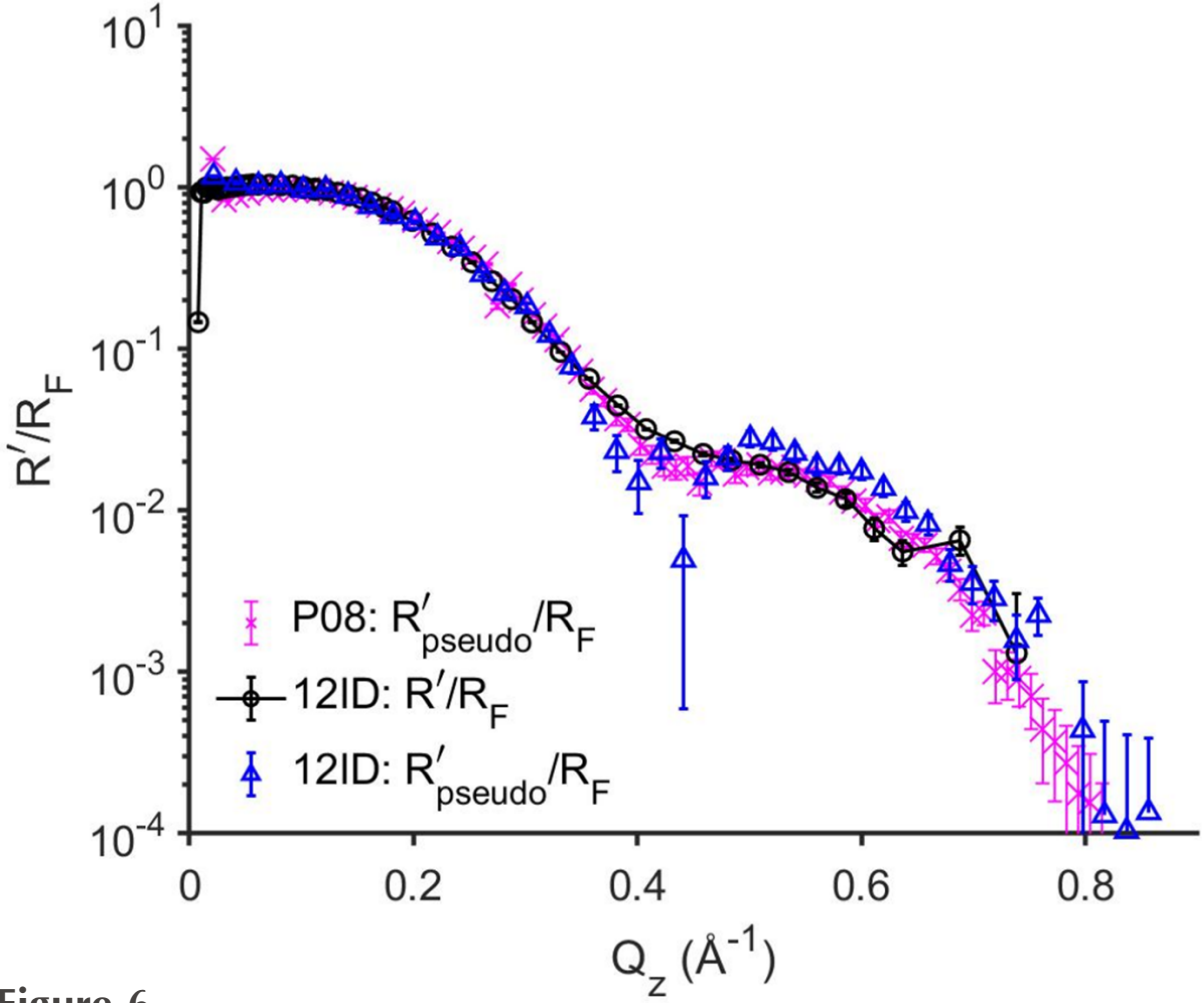
GIXOS-derived 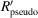 (blue triangles) and XRR-measured *R*′ (black circles) from the same Gibbs adsorption layer of CTAB (γ = 45 mN m^−1^) at the air–water interface at 292 K from a 0.6 m*M* solution (12ID, NSLS-II). Separately obtained 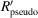 at P08 from the same system is included for reference (magenta crosses). 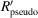 are derived using the *Q*_*z*_-dependent δ*Q*_*xy*,R_ of the rectangular angular opening used in the XRR measurement (Δβ = ±0.5 mrad, Δ2θ = ±0.5 mrad). Note that, due to the impurity, the reflectivity fringes here differ slightly from the reflectivity of the Gibbs layer from a highly purified CTAB solution (Sloutskin *et al.*, 2022 ▸).

Measurements on the free surface of a CTAB solution at a concentration below its critical micelle concentration were carried out using both reflectivity methods on the same sample. Here a Gibbs adsorption monolayer is formed that gives rise to a modulated reflectivity profile which differs from the monotonically decaying profile of simple liquids (Fig. 6 ▸). Particularly relevant to the present analysis is that the monolayer exhibits a bending modulus smaller than 3*k*_B_ *T* so that equations (8) and (9) are applicable. This is supported by the fact that the diffuse scattering 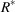 follows the CWM ( 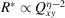 , Section S11). CTAB surface reflectivity results are first presented for measurements performed at the OPLS endstation of 12ID at NSLS-II. Taking into account the XRR background subtractions (see Section 3), we compare the off-specular subtracted pseudo-reflectivity 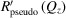 with its XRR-measured *R*′(*Q*_*z*_) [equations (14) and (15), Fig. 6 ▸] measured on the same sample. The results show reasonable agreement, with a minor deviation in the high-*Q*_*z*_ region (>0.4 Å^−1^), between the 12ID 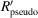 (blue triangles) and *R*′ (black circles). Additional measurements, carried out at the lower-background P08 GIXOS instrument (magenta crosses), provide 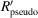 that agrees even better with the XRR-measured *R*′ than with the 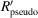 obtained at 12ID. Overall, the consistency between 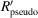 and *R*′ from the Gibbs layer of CTAB validates the applicability of the GIXOS-XRR method to thin surface films that exhibit weak stiffness.

## 6. Discussion and summary

The theoretical expression for the specular XRR from a liquid surface contains a term related to the capillary wave surface roughness and a second term related to the Fourier transform of the instrinsic SLD profile along the surface normal [equation (4)] (Pershan, 2000 ▸; Braslau *et al.*, 1988 ▸). A principal aim of XRR studies on liquid surfaces is to obtain the surface-normal structure by least-squares fitting analysis using physically motivated forms of the structure factor. Whereas specular XRR experiments are carried out by measuring reflected intensities under the condition where the exit scattering angle is set to the incident angle, we have shown above how *R*_pseudo_ can be obtained from the diffuse scattering around the specular reflection at a single, fixed incident angle, using GIXOS measurement. As with specular XRR, *R*_pseudo_ allows one to use the identical approach for obtaining the surface-normal structure from liquid surfaces.

To validate the approach of using GIXOS-measured diffuse scattering to obtain the reflectivity, we first investigated the liquid samples at different surface tensions, namely pure water and a water/ethanol mixture. These are simple interfaces without surface layering where their surface tensions and diffuse scattering are well documented (Schwartz *et al.*, 1990 ▸; Sanyal *et al.*, 1991 ▸; Vazquez *et al.*, 1995 ▸; Shpyrko *et al.*, 2004 ▸; Vaknin *et al.*, 2009 ▸). Their derived *R*_pseudo_ at several off-specular positions *Q*_*xy*_|_β = 0_ all agree with the specular reflectivity predicted by the CWM (Fig. 5 ▸), showing the robustness of the method. The measured diffuse scattering indeed shows the expected CWM-predicted 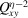 behavior at all *Q_z_* (Fig. 3 ▸), where the η parameter is computed using the measured surface tension, temperature and *Q_z_* value. This GIXOS-XRR method and its applicability to more complex liquid surfaces are validated by GIXOS and conventional XRR measurements on the same CTAB Gibbs monolayer, an equilibrium monolayer that forms at the air–liquid interface of a bulk solution of CTAB.

Moreover, our results also show that this GIXOS-XRR method provides a reflectivity curve that extends beyond 11 orders, a dynamic range that is not achievable using conventional specular XRR (Figs. 4 ▸ and 6 ▸). This higher *Q_z_* range permits structural analysis with better spatial resolution. This larger dynamic range is due to two features of the GIXOS measurement. Firstly, the GIXOS-XRR method uses fully the extensive wings of the surface diffuse scattering around the specular reflection, whereas diffuse scattering reduces the reflection intensity measured by the specular XRR (Shpyrko *et al.*, 2004 ▸). This allows GIXOS measurements to enlarge the effective detection area to collect more of the diffuse scattering, which improves the statistics [Fig. 2 ▸(*a*)]. Secondly, in the GIXOS case, the bulk scattering depth is ∼100 Å (∼ 2/*Q*_c_) for the total reflection condition (incident angle is below the critical angle). (The 1/*e* penetration depth of the X-ray intensity under the total reflection condition is 2/*Q*_c_ when the incident angle is 85% of the critical angle, and 1/*Q*_c_ when the incident angle approaches zero.) In the case of the specular XRR, the bulk scattering depth is more than 1000× larger and is set by the attenuation length of the subphase material. Hence the bulk scattering contribution relative to the surface scattering signal is much smaller with GIXOS measurements compared with specular XRR measurements, especially in the high-*Q_z_* region. For instance, for the water surface at *Q_z_* = 0.8 Å^−1^, the surface diffuse signal using the GIXOS method is about 10% of the bulk scattering signal [Section S7, Fig. S7(*a*)], making it still possible to resolve the surface signal with sufficient statistics after the bulk scattering subtraction.

The GIXOS-XRR method presents several advantages over conventional specular XRR: (i) The dynamic range of *R*_pseudo_ from the GIXOS-XRR method is larger, allowing the reflectivity to be obtained reliably up to a higher *Q_z_*. (ii) With GIXOS, the reflectivity over the entire *Q_z_* range is obtained in a single shot with a fixed footprint. This enables kinetic and *operando* measurements, and also pump–probe schemes. This method provides the same illuminated region for all *Q_z_*, and avoids variation of the X-ray footprint with *Q_z_* which is inherent with specular XRR. Moreover, there is much less beam damage under grazing incidence conditions as the flux per unit surface area is reduced by about a factor of 100 at the highest *Q_z_* values compared with specular XRR. (iii) The GIXOS setup is simpler than the specular XRR setup. Whereas crystal reflection optics are required for specular XRR to deflect the beam down over a range of α (Als-Nielsen & Pershan, 1983 ▸; Schlossman *et al.*, 1997 ▸; Honkimäki *et al.*, 2006 ▸; Murphy *et al.*, 2014 ▸), a bounce-down mirror is sufficient for GIXOS measurements. Further, the range of vertical sample motion is much reduced for GIXOS measurements. (iv) Whereas with specular reflectivity the in-plane resolution δ*Q*_*xy*,R_ varies with *Q_z_*, it is possible to use a fixed in-plane resolution for all *Q_z_* to calculate *R*_pseudo_. This fixed resolution gives rise to a *Q_z_*-independent σ_R_. (v) Excellent background shielding and reduction of parasitic small-angle X-ray scattering background are achievable with GIXOS measurements. With a bounce-down mirror it is easy to remove most of the air scattering from the beam flight paths. In addition, a small narrow guard slit is introduced in the sample chamber to further reduce parasitic small-angle scattering before the sample.

## 7. Related literature

The following additional references are cited in the supporting information: Baker *et al.* (2010 ▸), Fukuto *et al.* (1998 ▸), Hura *et al.* (2000 ▸), Mechler *et al.* (2010 ▸), Orthaber *et al.* (2000 ▸), Shpyrko *et al.* (2003 ▸) and Tristram-Nagle & Nagle (personal communication).

## 8. Conclusion

In this paper we provide mathematical expressions, based on the capillary wave model of liquid surfaces, to reconstruct the reflectivity curve from the diffuse scattering results acquired by grazing incidence X-ray off-specular scattering measurements at a fixed incident angle. This method provides an alternative technique to measure the surface-normal structure, other than conventional specular XRR, while still allowing the use of standard XRR analysis software tools. This method also provides a better signal–noise ratio, faster acquisition, less beam damage and a larger *Q_z_* range compared with conventional XRR. The faster acquisition enables time-resolved surface structure analysis. The GIXOS-XRR method utilizes a simple experimental setup, one suitable for many existing focused synchrotron beamlines by the addition of a few additional components. While the present work does not consider the role of bending rigidity, these effects will be included in a subsequent paper. This not only expands the use of the GIXOS-XRR method to stiffer surface layers, it also provides an explicit method for calculating the bending rigidity on liquid surfaces.

## Supplementary Material

Supporting information. DOI: 10.1107/S1600576724002887/xx5044sup1.pdf

## Footnotes

1 (i) The cut-off *Q*_l,c_ in 〈*h*(*r*_*xy*_)*h*(0)〉 cancels out during the computation of dσ/dΩ. (ii) The following expression of dσ/dΩ does not include the effect of the limited coherent length ξ_coh_ of the synchrotron beam (typically on the order of tens of µm). With the limited coherent length, the Fourier transform is only integrated up to ξ_coh_ in *r_xy_*, and may result in a broadening of the singularity of dσ/dΩ at *Q_xy_* = 0 to a width of 1/ξ_coh_ (HWHM) (Dutta & Sinha, 1981 ▸; Sinha *et al.*, 1988 ▸). This broadening is negligible for most XRR and GIXOS measurements as the resolution is dominated by the solid-angle opening of the detector and is much broader than 1/ξ_coh_ [see equation (3)].

2 In Schwartz’s study, the 0.8 mm-high slit opening at 600 mm from the sample gives Δβ = 1.33 mrad and  = 1.7 × 10^−4^ Å^−1^ at *Q_z_* = 0.5 Å^−1^, the largest *Q_z_* in their study (λ = 1.259 Å). In Vaknin’s study, the 1.5 mm-high slit opening at 750 mm from the sample at 16.2 keV gives δ*Q*_*xy*,R_ = 2.5 × 10^−4^ Å^−1^ at *Q_z_* = 0.5 Å^−1^.

3 Schwartz *et al.* (1990 ▸) report σ_0_= 0 Å using *Q*_max_ = (π/1.4) Å^−1^, while Vaknin *et al.* (2009 ▸) report the same using *Q*_max_ = (π/1.93) Å^−1^. According to equation (5) using the correct *a*_m_ = 3.1 Å, their results correspond to σ_0_ = 0.85 and 0.63 Å, respectively.
