# Supplementary material for: Reconstructing the reflectivity of liquid surfaces from grazing incidence X-ray off-specular scattering data
Source: J Appl Crystallogr. 2024 May 17;57(Pt 3):714–27. doi: 10.1107/S1600576724002887 (PMC11151673; doi:10.1107/S1600576724002887)
Supplement: Supplementary file 1 [file j-57-00714-sup1.pdf]

JOURNAL OF APPLIED CRYSTALLOGRAPHY

Volume 57 (2024)

Supporting information for article:

Reconstructing the reflectivity of liquid surfaces from grazing-incidence X-ray off-specular scattering data

Chen Shen, Honghu Zhang and Benjamin M. Ocko

Supporting information

S1. Effect of the transmission coefficient and the footprint broadening

The transmission coefficient $t(\beta)$ for the vertical scattering angle β is (Feidenhans'l, 1989)

$$|t(\beta)|^2 = \left| \frac{2\left(\beta/\alpha_c\right)}{\beta/\alpha_c + \sqrt{\left(\beta/\alpha_c\right)^2 - 1 - \frac{2\beta_{abs}}{\alpha_c^2} i}} \right|^2$$

where $\beta_{abs} = 10^{-9}$ and $\alpha_c = 0.081^{\circ}$ are the imaginary part of the refractive index and the critical angle, respectively, both of water for 15 keV X-ray beam. The transmission coefficient $t(\alpha)$ of the incident beam follows the same equation in which the angle β is replaced by α.

The scattering comes from a 57 mm long and 0.25 mm wide footprint instead of a point source. Hence each pixel on the detector corresponds to a range of β and 2θ as the detected photon can come from anywhere on the footprint. This has two impacts. Firstly, for each pixel $t(\beta)$ must be averaged over the allowable β. Secondly, the $\Delta\beta$ and $\Delta 2\theta$ for each pixel in Equation 8 and Equation 9 are also enlarged, compared to the values assuming the scattering from a point source.

In Figure S1, the result of using Equation 10 (red star) with neither $t(\beta)$ contribution nor the footprint broadening effect is compared to the effect of including $t(\beta)$ contribution (blue circle), and the further effect of footprint broadening (black cross). It shows that $t(\beta)$ only affects the region near the critical angle (Q_z < 0.03 Å^{-1}) where only 1st two data points deviate from $\lim_{Q_z \to 0} R_{pseudo}/R_F = 1$. It also shows that the results with and without the footprint

broadening are indistinguishable. Since the results overlap, the footprint broadening is neglected in the analysis.

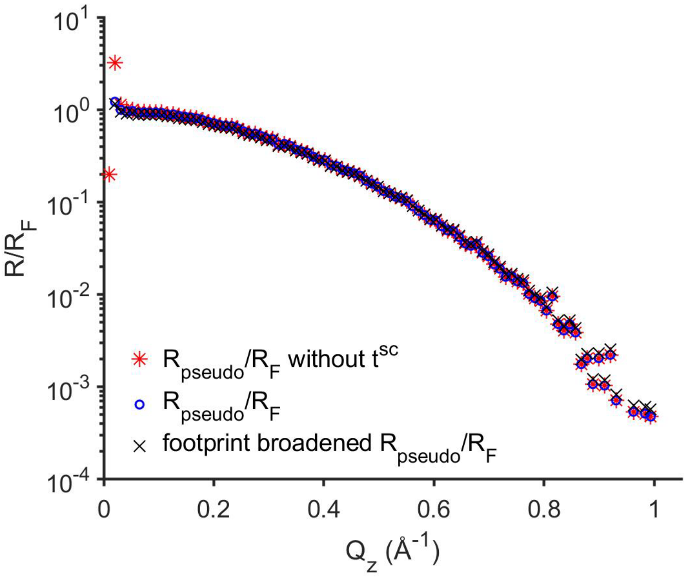

Figure S1 Effect of the corrections on the reconstructed pseudo reflectivity from the diffuse scattering around the specular reflection. Pseudo reflectivity without transmission coefficient $t(\beta)$ of the scattered beam, with $t(\beta)$, and with further broadened $\Delta\beta$ and $\Delta 2\theta$ by 57 mm long, 0.25 mm wide footprint are depicted by different symbols.

S2. Effects of the angular resolution on the diffuse scattering data

Figure S2 shows that increasing Q_{xy}-resolution, by enlarging angular resolution $\Delta\Omega$, improves the statistics of the diffuse scattering around the specular reflection more efficiently than improves the statistics of the specular reflectivity. The curves are calculated for water surface using the Equation 6 and Equation 4, respectively. The diffuse scattering around the specular reflection is calculated for GIXOS geometry with an incident angle at 85% of the critical angle at 15 keV. The angular resolution of the curves on the right is 20× broader than the resolution of the curves on the left by using 20× larger $\Delta 2\theta$ and the same $\Delta\beta$. The diffuse intensity around the specular reflection can be increased proportionally by broadening the resolution (20× increase), whereas the specular reflectivity at Q_z =1 Å^{-1} is only increased by a factor of two.

(a) (b)

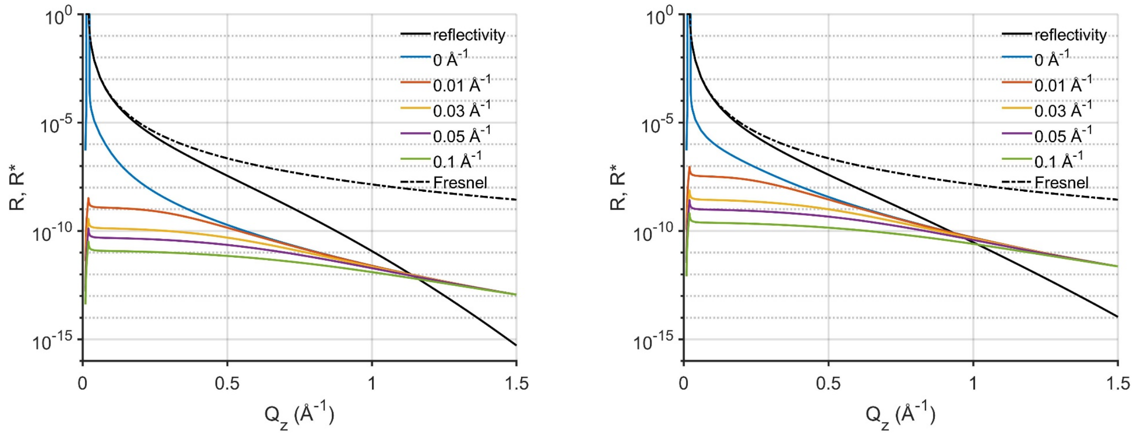

Figure S2 Simulated diffuse scattering around the specular reflection under GIXOS geometry from water at 293 K with $\Delta 2\theta$ of 0.004° (a) and of 0.08°(b). The two openings correspond to Q_x-resolution (HWHM) of ±2.7×10^{-4} Å^{-1} and ±55.3×10^{-4} Å^{-1}, respectively. The incident angle and energy are respectively set to 0.07° (85% of the critical angle of the air-water interface) and 15 keV. $\Delta\beta$ is 0.08°, that corresponds to Q_y-resolution (HWHM) of ±5.5×10^{-4} Å^{-1} at the largest Q_z (1.5 Å^{-1}). Diffuse scattering R^* at different off-specular positions $Q_{xy}\big|_{\beta=0}$ are color-coded as labelled. Note that the blue curves are the diffuse scattering in the plane of incidence ($Q_{xy}\big|_{\beta=0}$ = 0 Å^{-1}, i.e. 2θ = 0°). Fresnel reflectivity and reflectivity for water under the same detector opening are entered as references.

S3. Beamstop design

The 0.5 mm-thick Tungsten beamstop (Figure S3) has a 0.1 mm-thick edge that sticks out by 0.1 mm from the surface where the total reflection X-ray beam (red arrow) hits. 0.1mm extra edge thickness helps reduce secondary scattering from the sidewise splashed X-ray beam from where the beam impacts the beamstop (Tristram-Nagle & Nagle).

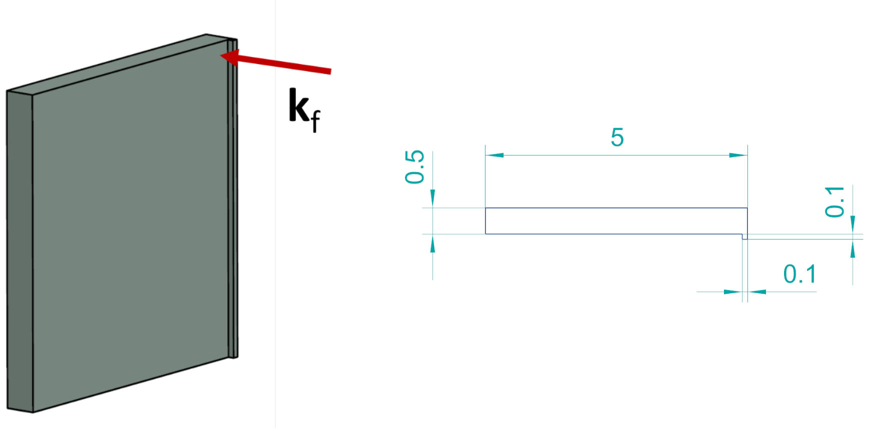

Figure S3 Isometric sketch of the beamstop (left) and its top-view drawing (right). The red arrow depicts the reflected X-ray beam from the interface with its wavevector $\boldsymbol{k_f}$, and the top corner next to the edge of the beamstop where this beam hits. The unit of the dimensions in the drawing is in millimeter.

S4. Transformation into (*β*, *2θ*) space, rebinning, grouping and correction

The values of the vertical (*β*) and the horizontal exit scattering angle (*2θ*) of each pixel of the raw detector images are calculated by the trigonometric relations with the known D_{det} = 561 mm, the horizontal and vertical pixel indices n_x and n_z and the pixel size *p* of 75 μm in both directions, and a small detector rotation error *ω* around y-axis (Figure 1a in main text, clockwise is defined as positive), which is normal to the frame:

$$2\theta = \operatorname{atan}\left(\frac{(n_x - n_{x,0}) \cdot p \cdot \cos\omega + [(n_z - n_{z,0}) \cdot p - D_{det} \cdot \tan\alpha] \cdot \sin\omega}{D_{det}}\right)$$

$$\beta = \operatorname{atan}\left(\frac{-(n_x - n_{x,0}) \cdot p \cdot \sin\omega + [(n_z - n_{z,0}) \cdot p - D_{det} \cdot \tan\alpha] \cdot \cos\omega}{D_{det} \cdot \cos(2\theta)}\right)$$

Here ($n_{x,0}$, $n_{z,0}$) is the pixel index coordinates of the incident beam. The detector rotation error is corrected such that the Yoneda peak at all *2θ* remains at the critical exit scattering angle $\beta_c = \operatorname{asin}\left(\frac{Q_c\lambda}{4\pi}\right)$. It is not necessary to correct the detector rotation errors around *x* and *z*-axis due to the mounting accuracy compared to the *β* and *2θ* deviation induced by these two rotations.

The data are then re-binned into an orthogonal (*β*, *2θ*)-space with step sizes Δ*β* = 0.008° and Δ*2θ* = 0.008°, and then grouped into step sizes Δ*β* = 0.08° and Δ*2θ* = 0.08°. The bin sizes of Δ*β* and Δ*2θ* are chosen to be about detector pixels resolution in both the vertical and the horizontal directions, and the grouping sizes are chosen to be 10× of that to enhance the signal noise ratio. The rebinning is performed since the (*β*, *2θ*) coordinates of the pixels in the data measured by a 2-dimentional detector at fixed position are not orthogonal due to a slight tilt (*ω* = 0.6°) of the detector mounting: pixels in the same row do not have the same *β*, and pixels in the same column do not have the same *2θ* (see Section 3 of the main text). Further, the axis are not linearly spaced as the step size is variable. For the grouping and background subtraction, it is convenient to rebin the data into an orthogonal (*β*, *2θ*) space with a linear spaced grid where the pixels in the same row have the same *β*, and pixels in the same column have the same *2θ*. During the re-binning, a pixel splitting scheme is utilized (Ashiotis *et al.*, 2015), whereby the intensity of a pixel in the original grid is distributed into the pixels in the new grid, that have the closest (*β*, *2θ*). This is described in the following. Note that at this stage of the data manipulation process, the intensity per pixel in the rebinned data has not yet

been corrected for the solid angle coverage $\Delta\Omega$ of the rebinned pixels, but remains for the solid angle coverage of the original detector pixel at the same (β, 2θ).

The indices in the original and rebinned space are named (i, j) and (i', j'), respectively. The i- and i'-direction corresponds to variation in the angle β. The rebinning is first performed in this direction for every column. The new linear β axis is set to cover from the minimal to the maximal β of the GIXOS dataset, with a step size $\delta\beta$. In the original coordinate, the *i-th* pixel in the *j-th* column is at an angle $\beta(i)_j$. In the β-rebinned coordinate, the *i'-th* pixels in all columns belongs to a unique angle $\beta(i')$.

The intensity as a function of β for each column must be kept the same before and after the rebinning. Hence, the intensity on the *i'-th* pixel in the *j-th* column in the β-rebinned coordinate needs to be calculated based on the β value of that pixel, since the pixels in the rebinned coordinate mostly do not have the β value of any pixel in the original data. To do this, the intensity $I_{raw}(i)_j$ on the *i-th* pixel in the *j-th* column of the original raw data needs to be distributed to the *i'-th* and the *(i'+1)-th* pixel in the *j-th* column of the β-rebinned coordinate, when $\beta(i') \le \beta(i)_j \le \beta(i'+1)$. The fraction $w(i;i')_j$ of this intensity that shall be distributed into the *i'-th* pixel in the *j-th* column of the β-rebinned data is:

$$w(i;i')_j = \begin{cases} \dfrac{\beta(i'+1) - \beta(i)_j}{\delta\beta}, & \beta(i') \le \beta(i)_j \le \beta(i'+1) \\ 0, & \beta(i)_j < \beta(i') \; or \; \beta(i)_j > \beta(i'+1) \end{cases}$$

and the fraction $w(i;i'+1)_j$ of this intensity to be distributed into the *(i'+1)-th* pixel in the *j-th* column of the β-rebinned data is

$$w(i;i'+1)_j = \begin{cases} 1 - w(i;i')_j, & \beta(i') \le \beta(i)_j \le \beta(i'+1) \\ 0, & \beta(i)_j < \beta(i') \; or \; \beta(i)_j > \beta(i'+1) \end{cases}$$

Using the two fractions, the intensity on the *i'-th* pixel of the *j-th* column of the β-rebinned data is then calculated as

$$I_{bin\beta}(i',j) = \frac{\sum_i \left[I_{raw}(i-1)_j \cdot w(i-1;i')_j + I_{raw}(i)_j \cdot w(i;i')_j \right]}{\sum_i \left[w(i-1;i')_j + w(i;i')_j \right]}$$

The 2θ value of the pixels in the rebinned coordinate also needs to be calculated, since the original grid is not orthogonal: pixels in the same column do not have the same 2θ, but their 2θ is correlated to the β-positions of the pixels (see Section 3 of the main text). The pixels in the *j-th* column of the rebinned coordinate now is at a different vertical position from any

pixel in the *j-th* column of the original grid. The $2\theta(i')_j$ of the *i'-th* pixel in the *j-th* column of the rebinned coordinate is calculated based on the 2θ of the *(i-1)-th* and the *i-th* pixel in the *j-th* column of the original data, when $\beta(i-1)_j \leq \beta(i') \leq \beta(i)_j$. This leads to a similar expression to the intensity calculation:

$$2\theta(i',j) = \frac{\sum_i \left[2\theta(i-1)_j \cdot w(i-1;i')_j + 2\theta(i)_j \cdot w(i;i')_j\right]}{\sum_i \left[w(i-1;i')_j + w(i;i')_j\right]}$$

The β coverage of each column in the original dataset is smaller than the coverage of the new β-axis, since the latter covers from the minimal to the maximal β of the whole 2D frame. The pixels outside of the measured β-range of that column are assigned as Not-a-Number (NaN). Moreover, if the step size $\delta\beta$ is set to be close to the averaged step size of the original dataset, the intensity of a few pixels within the measured β-range of that column may not have been calculated, since no pixel in the original grid of the same column index has a β value that is in between the β values of the two neighbours of these pixels. The intensity in such pixels is linearly interpolated from the closest neighbouring two pixels on the two sides.

After the β rebinning, pixels in each row have the same β, while the pixels in each column still do not have the same 2θ. Therefore the data is rebinned in 2θ for every row. Here only the intensity is distributed into the new grid, following the same manner but along 2θ. Similar to the β-rebinning, a new linear 2θ axis is set to cover from the minimal to the maximal 2θ of the GIXOS dataset, with a step size $\delta 2\theta$. In the β-rebinned grid, the *j-th* pixel in the *i'-th* row is at an angle $2\theta(j)_i$. In the (β, 2θ)-rebinned, the *j'-th* pixels in all rows belongs to an angle $2\theta(j')$. Hence, for the *i'-th* row of the β-rebinned data, the fraction $u(j;j')_{i'}$ of the intensity on the *j-th* pixel that shall be distributed into the *j'-th* pixel of the (β, 2θ)-rebinned data is:

$$u(j;j')_{i'} = \begin{cases} \dfrac{2\theta(j'+1) - 2\theta(j)_{i'}}{\delta 2\theta}, & 2\theta(j') \leq 2\theta(j)_{i'} \leq 2\theta(j'+1) \\ 0, & 2\theta(j)_{i'} < 2\theta(j') \; or \; 2\theta(j)_{i'} > 2\theta(j'+1) \end{cases}$$

and the fraction $u(j;j'+1)_{i'}$ of this intensity to be distributed into the *(j'+1)-th* pixel is

$$u(j;j'+1)_{i'} = \begin{cases} 1 - u(j;j')_{i'}, & 2\theta(j') \leq 2\theta(j)_{i'} \leq 2\theta(j'+1) \\ 0, & 2\theta(j)_{i'} < 2\theta(j') \; or \; 2\theta(j)_{i'} > 2\theta(j'+1) \end{cases}$$

Eventually, the intensity on the *j'-th* pixel of the *i'-th* row of the final (β, 2θ)-rebinned data is

$$I_{bin}(i',j') = \frac{\sum_j \left[I_{bin\beta}(j-1)_{i'} \cdot u(j-1;j')_{i'} + I_{bin\beta}(j)_{i'} \cdot u(j;j')_{i'}\right]}{\sum_j \left[u(j-1;j')_{i'} + u(j;j')_{i'}\right]}$$

Thereafter, every 10 pixels in both the vertical (β) direction and the horizontal (2θ) direction are grouped to enhance the signal-noise ratio, with the final resolutions $\Delta\beta$ = 0.08° and $\Delta 2\theta$ = 0.08°.

The intensity in the rebinned and grouped grid is now corrected for final resolution $\Delta\beta$ = 0.08° that is defined by the final β-step size. This correction is necessary because the resolution $\Delta\beta_0$ for each original detector pixel varies with β, for a fixed flat detector with evenly distributed pixel size. The corrected data $I(\beta)|_{2\theta}$ is obtained by normalising the grouped $I_g(\beta)$ to the solid angle projection $s(\beta, \delta\beta)/s(0, \delta\beta) = [\sin(\beta + \Delta\beta) - \sin\beta]/\sin(\Delta\beta)$ of the pixel facing the angle β:

$$I(\beta)|_{2\theta} = I_g(\beta)\big|_{2\theta} \cdot \frac{s(0, \Delta\beta)}{s(\beta, \Delta\beta)}$$

Here we do not correct the intensity for the resolution $\Delta 2\theta$ since the variation of $\Delta 2\theta$ of the original detector pixel at different β positions is negligible in the range of our data acqusition (2θ < 2.4° and β < 7°).

S5. Raw detector images

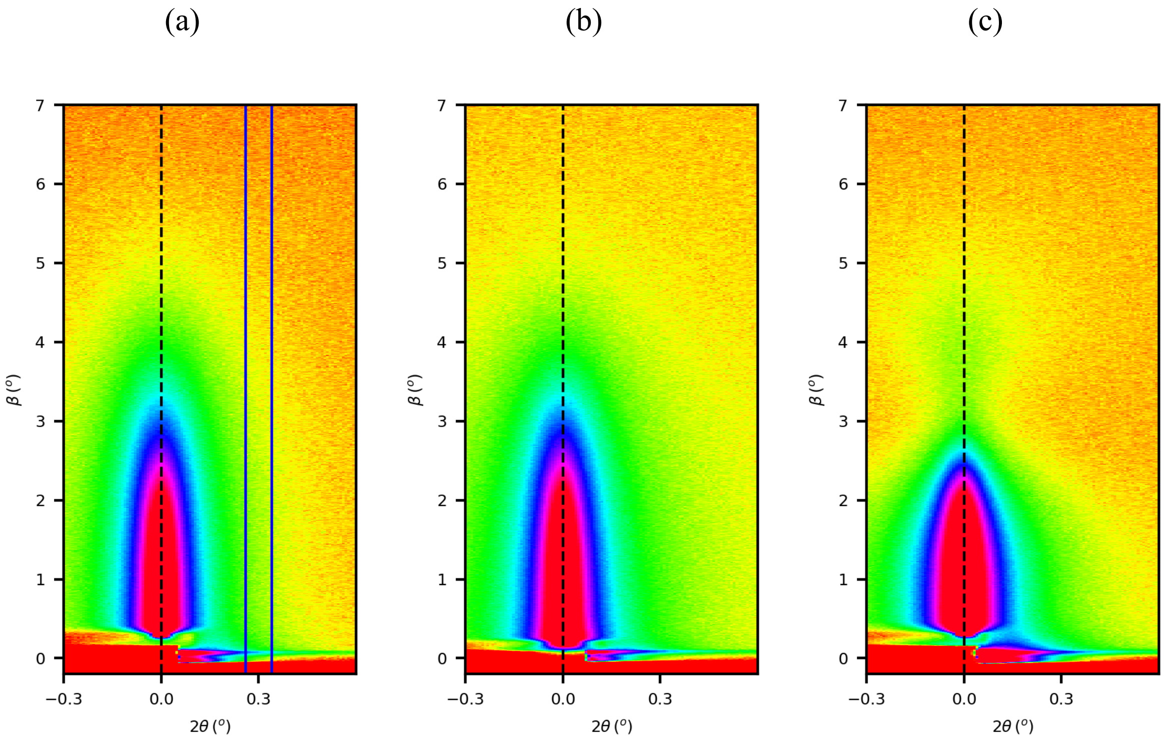

Figure S4 Raw detector data from P08 measurements up to 2θ = 0.6° from the surfaces of (a) water, (b) a 10% ethanol in water mixture, and (c) a Gibbs layer formed from a bulk solution of 0.6 mM CTAB in water. Black dashed lines mark the plane of incidence (2θ = 0). The two blue lines in (a) depict an off-specular region centred at 2θ = 0.3° ($Q_{xy}|_{\beta=0}$ = 0.04 Å^{-1}) with a horizontal angular opening $\frac{\Delta 2\theta}{2}$ = ±0.04° that is used to obtain the GIXOS profile R^* for this $Q_{xy}|_{\beta=0}$. Within this region, the intensity is binned along 2θ, and every 0.08° along β. R_{pseudo} is calculated using Equation 8 with $\Delta 2\theta$ = 0.08° and $\Delta\beta$ = 0.08°.

S6. Reconstructed pseudo reflectivity from the diffuse signal at different off-specular positions

Figure S5 shows (a) the GIXOS-measured diffuse scattering around the specular reflection from the water surface measured at different off-specular positions, and (b) R_{pseudo}/R_F reconstructed by Equation 10 from these diffuse scattering data. In (a), as a guide to eye, calculated R/R_F curves using different Gaussian roughness between 1.8 Å and 3.4 Å are entered. In (b) the only varying parameters are the off-specular position $Q_{xy}|_{\beta=0}$, and accordingly the Q_{xy} value at each β. The values of the off-specular position $Q_{xy}|_{\beta=0}$ for the color-coded data are given in the legend in (b), and $Q_{xy}|_{\beta=0}$ = 0 Å^{-1} position refers to the place of incidence. The diffuse scattering at different off-specular positions show different Q_z dependency. Near the plane of incidence the diffuse scattering (a, blue, red) does not follow R/R_F drop, and at larger $Q_{xy}|_{\beta=0}$ (the other three), the intensity drop apparently follows R/R_F courses with smaller interfacial roughness than the phenomenological Gaussian roughness predicted by CWM (σ_R = 2.8 Å for water at 295 K). Hence, directly fitting the GIXOS-acquired R^* by $R^* \propto R/R_F$ (that is equivalent to setting $r \propto 1/R_F$) will provide an unphysically low roughness (Oliveira *et al.*, 2010; Shen *et al.*, 2022). In contrast, R_{pseudo}/R_F from different off-specular positions all follow the CWM predicted R/R_F for water (295 K, 73 mN/m), and give σ_R = 2.8 Å and this agrees with the previous XRR studies (Schwartz *et al.*, 1990).

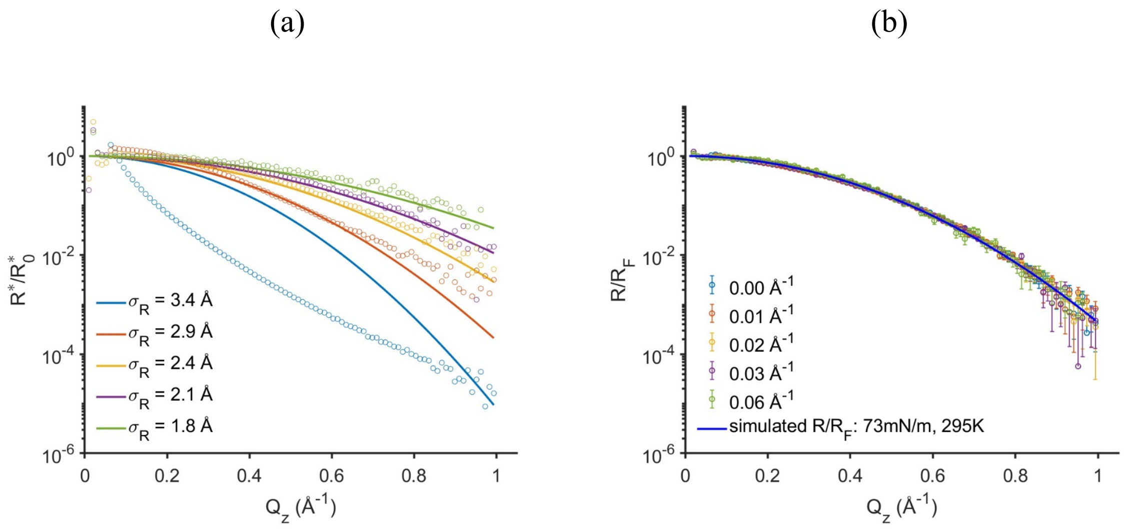

Figure S5 Diffuse scattering data measured by GIXOS and the pseudo reflectivity from water surface at 295K. (a) diffuse scattering from different off-specular $Q_{xy}\big|_{\beta=0}$ positions (labelled). They are normalized to the Q_z-independent factor $R_0^* = \frac{|t(\alpha)|^2 \rho_{b,\infty}^2 k_B T \Delta\beta \Delta 2\theta}{\sin\alpha \cdot \gamma \cdot Q_{xy}^2}$. Fresnel reflectivity curves modified by assumed phenomenological roughness are entered as solid lines. (b) pseudo reflectivity with an in-plane resolution $\delta Q_{xy,R}$ of 2×10^{-4} Å^{-1}, derived from the diffuse scattering data in (a), and the R/R_F curve (blue solid line) predicted by CWM for a surface with 73 mN/m, 295 K, σ_0 = 0.4 Å.

S7. Background contributions in the diffuse scattering data

The quality of the pseudo reflectivity reconstruction depends on the ratio between the surface diffuse scattering signal and the background signals, and this requires an accurate measurement of the latter. Therefore, the GIXOS instrument design needs to suppress the background as much as possible. The background signals come from the following three contributions: (1) the scattering signals from the windows hit by the beam, i.e. the exit window of the incident beam path, the entrance and exit window of the sample enclosure, (2) scattering along the beam path through the helium atmosphere, and (3) the liquid bulk structure factor.

The first background contribution, namely the Kapton window scattering, appears as rings at a relatively wide angle, i.e. between 0.3 and 0.6 $Å^{-1}$. This can be efficiently suppressed by proper shielding or collimation. A narrow vertical guard slit about 30mm after the sample chamber entrance window and before the sample is sufficient to block the Kapton window scattering. In addition, a beamstop before the exit window will prevent the generation of scattered background from the exit window. If the installation of a guard slit and a beamstop inside the sample chamber is not possible, a simple alternative to reduce the parasitic background is a set of collimation slits after the exit window and before the detector[*] that defines the off-specular angle to be measured (Fradin *et al.*, 2000; Dai *et al.*, 2011). Proper opening sizes and off-specular angle will permit only the beam scattered from the footprint region to pass but nothing else. The second background contribution is strongest at the low angle but still contributes to a significant relative portion in wide angle where the diffuse scattering signal from the liquid surface is weak. This instrument scattering can be measured by lowering the sample to let the incident beam directly hit the beamstop.

The third background contribution is the bulk scattering of the liquid sample (e.g. from water) and in principle it should be isotropic and this implies that there is little variation at different azimuth angles. Along each azimuthal direction, this bulk scattering is almost constant at low

---

[*] In this configuration, a vertically mounted 1-dimensional position sensitive detector would be sufficient to obtain a β dependent GIXOS profile. With an area detector, the detector slit can be substituted with a virtual slit by defining a 0.5 mm wide region of interest on the area detector.

Q, and this intensity at low Q limit is proportional to the isothermal compressibility κ_T of the liquid (Orthaber *et al.*, 2000; Fradin *et al.*, 2000):

$$\lim_{Q\to 0} I_{bulk}(Q) \propto \frac{d\sigma_{bulk}}{d\Omega} \approx \frac{A_0}{\sin\alpha} \cdot \rho_{b,\infty}^2 |t(\alpha)|^2 |t(\beta)|^2 \cdot \frac{k_B T \kappa_T}{2\mathrm{Im}(Q_z')}$$

σ_{bulk} and $Q_z' = \frac{2\pi}{\lambda}\left(\sqrt{\sin^2\beta - \sin^2\alpha_c - 2\beta_{abs}i} + \sqrt{\sin^2\alpha - \sin^2\alpha_c - 2\beta_{abs}i}\right)$ are respectively the bulk scattering cross section and the surface normal scattering vector in the liquid (note that Q_z' is not Q_z – the surface normal scattering vector above the surface). $\mathrm{Im}(Q_z')$ is the imaginary part of Q_z' and its reciprocol represents the scattering depth defined by α and β (Feidenhans'l, 1989). The bulk scattering (bulk structure factor) starts to increase significantly from Q ~ 0.8 Å^{-1} and its first maximum appears at a much larger Q (~ 2 Å^{-1}) that is related to the intermolecular spatial correlation (Hura *et al.*, 2000). In the Q range up to 1.4 A^{-1}, the relevant Q range for GIXOS-XRR measurements, the intensity monotonically increases with increasing Q. Figure S6a shows the GIXOS-measured scattering profile along different azimuths, after the subtraction of the instrument contribution. After the instrument background subtraction, the remaining GIXOS scattering signal consists of the surface diffuse scattering R^* around the specular reflection and this bulk scattering I_{bulk}. Near the specular axis ($Q_{xy} < 0.3$ Å^{-1}, $2\theta < 2.4°$), the surface diffuse scattering around the specular reflection is strong due to the $Q_{xy}^{\eta-2}$ dependency ($\eta < 2$), hence causes the high intensity in the Q range smaller than the arrow positions. At higher Q where $Q_{xy} > 0.3$ Å^{-1}, the surface diffuse scattering contribution is negligible and mainly the bulk scattering contributes. In this Q range, the almost identical intensity dependency on Q at different azimuthal directions are due to the isotropic behaviour of the bulk water scattering. Hence, the 2D detector that covers sufficient Q range at $Q_{xy} > 0.3$ Å^{-1} permit a direct measurement of the bulk scattering for subtraction.

When a 2D detector with sufficient Q-coverage is used (e.g. P08 data), the GIXOS profiles at different azimuthal directions, after the chamber background subtraction, can be utilized to obtain the bulk scattering contribution of the sample that covers the whole Q range needed for the GIXOS-XRR method. The principle is explained here. First of all, the Q value where $Q_{xy} = 0.3$ Å^{-1} varies with the azimuthal direction. Hence azimuthal averaging of all the profiles for the range $Q_{xy} > 0.3$ Å^{-1}, while avoiding the horizon where Yoneda peak is, will provide a bulk scattering data covering a Q range from 0.3 Å^{-1} to 1.4 Å^{-1}, the largest Q value accessible for the detector (Figure S6b). This is possible due to the isotropy of the bulk

scattering. An exponential growth function was used for fitting the I_{bulk} *versus* Q phenomenologically (Figure S6b, dash-dot line).

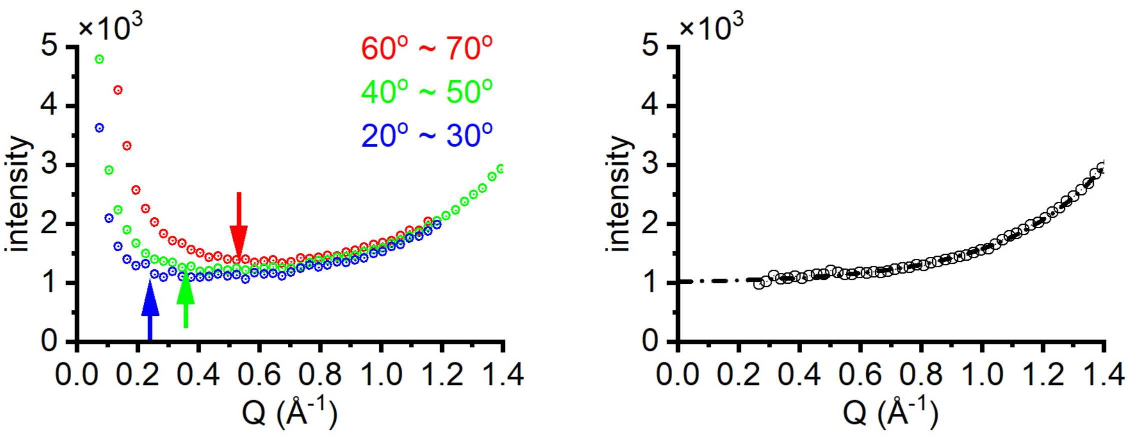

Figure S6 GIXOS intensity as a function of $Q = \sqrt{Q_{xy}^2 + Q_z^2}$ after the chamber background subtraction, from the water surface sample measured on the P08 instrument. (a) Scattering profiles (color-coded) along three azimuthal direction χ ranges with respect to the horizon (χ = 0°). The plane of incidence is at 90°. They are all averaged over an azimuthal range of ±5°. Arrows mark the Q value at 2θ = 2.4° ($Q_{xy}|_{\beta=0}$ = 0.3 Å^{-1}) for the azimuthal direction with the same color code in the legend. (b) The azimuthal averaged profile from 2θ > 2.4°, β > 0.2° and its fit (dashed line): $I_{bulk} = 994.4 + 27.9 \cdot \exp(3.04Q)$.

Figure S7 shows the raw GIXOS data from the water surface and from the chamber background measurement at $Q_{xy}|_{\beta=0}$ = 0.03 Å^{-1}, aforementioned fit bulk scattering profile I_{bulk} at the same $Q = \sqrt{Q_{xy}^2 + Q_z^2}$ as the water surface data, and the diffuse scattering profile obtained from the subtraction of the chamber and the bulk scattering data, which was used for the calculation of the pseudo reflectivity. The water raw data shows a clear small angle scattering intensity from the chamber background at low angle, in addition to the Yoneda peak contribution, and can be subtracted away by using the chamber background. The chamber background subtracted water data (black solid line) still remains ~3000 intensity at Q_z > 0.8 Å^{-1}. This is due to the contribution of the water bulk scattering that is to be subtracted away by using fit bulk scattering profile I_{bulk} ((b), purple dashed line). After the subtraction of I_{bulk}, the remaining intensity ((b), circles) is the diffuse scattering around the

specular reflection, and shows a monotonically decreasing intensity with increasing Q_z down to 100 at Q_z = 1 Å^{-1} ((b), insert).

(a) (b)

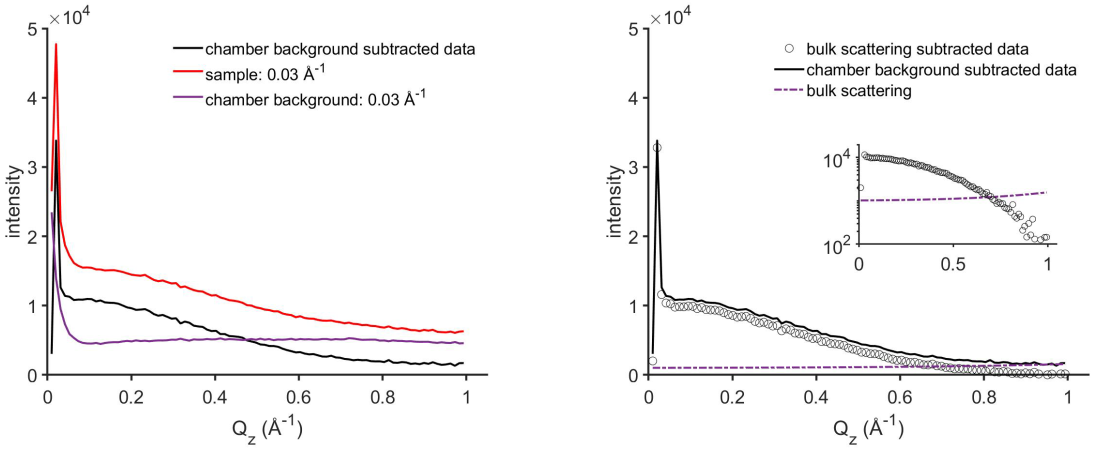

Figure S7 GIXOS profile from the water measurement and its contributions. (a) Raw GIXOS profile of water at $Q_{xy}\big|_{\beta=0}$ = 0.03 Å^{-1} (red line), the chamber background at the same $Q_{xy}\big|_{\beta=0}$ (purple solid line) and the GIXOS profile after the chamber background subtraction (black line). (b) The GIXOS profile after the chamber background as in (a), the fit bulk scattering profile I_{bulk} (purple dashed line, see Figure S6b), and the diffuse scattering around the specular reflection from the water surface that is obtained from the subtraction of the chamber background and the bulk scattering (black circle). The insert presents surface diffuse scattering and the bulk scattering fit in logarithmic scale.

The 1-dimensional GIXOS profile obtained with the two slit geometry at 12ID-OPLS (Dai et al., 2011) does not permit this way of extracting the precise bulk scattering contribution. This is because only the high Q_z-range (> 0.75 Å^{-1}) is dominated by the bulk scattering contribution and the surface the interfacial structure factor fringes and the capillary wave diffuse scattering signal dominates the majority of the data (Sloutskin et al., 2022) (see above and Figure S7). Its bulk scattering contribution, together with any residue background after the instrument signal subtraction, is phenomenologically estimated as a lump-sum by a linear fit of the high Q_z-range data (> 0.75 Å^{-1}), and extrapolated to the whole Q_z-range for the subtraction. The error from this way of estimating the bulk scattering is negligible in the low Q_z-range (< 0.65 Å^{-1}) where the modulation of the reflectivity by the structure factor fringes (Sloutskin et al., 2022) and the capillary wave diffuse scattering are dominant (see above and

Figure S7). Hence it is suitable for our purpose of using the modulated part of the reflectivity curve to compare the two reflectivity methods.

S8. Background subtraction in conventional specular reflectometry

In typical specular XRR measurements the background is obtained by measurements of the off-specular signal. Prior to the use of 2D detectors, the background has been obtained by displacing the detector horizontally by a distance corresponding to the resolution width, typically of order 0.1-0.4 °. In the case of 2D detectors, the background is typically obtained by measuring an equivalent number of off-axis pixels as in the specular channels. The XRR-measured reflectivity is R' given by Equation 13 and Equation 14. In either case, this approach over estimates the background since the signal measured at $2\theta_{bkg}$ also contains remnants of the surface diffuse scattering due to the $Q_{xy}^{\eta-2}$ tails (Shpyrko *et al.*, 2004). From Equation 13, the relative deviation of R' from R is r. Hence, the specular signal is reduced at large Q_z since r is always positive and increasing with Q_z. This overestimates the effective broadening of the density profile and hence it is not appropriate to directly compare our water pseudo reflectivity results presented above with earlier specular XRR measurements on water (Braslau *et al.*, 1988; Murphy *et al.*, 2014).

In the late 1990s (Pershan, 2000; Fukuto *et al.*, 1998; Tostmann *et al.*, 1999; Schwartz *et al.*, 1990), it was realized that R' should be directly compared with the difference between the calculated R and R^* values since the bulk scattering background should be virtually the same for small offsets in Q_{xy}, given by Equation 14. Studies of the water surface (Shpyrko *et al.*, 2004), for Langmuir monolayer (Fukuto *et al.*, 1998) and the liquid metal surfaces (Shpyrko *et al.*, 2003; Tostmann *et al.*, 1999) and gold-silicon eutectic (Mechler *et al.*, 2010) accounted for the background using Equation 14 and found good agreement with the CWM. It is for this reason, that we directly compare our water results with those of Shpyrko rather than the other measurements since those measurements didn't fully account for the background.

S9. Comparison of the normalisation of specular reflection and of diffuse scattering intensity

The intensity normalisation of the diffuse scattering signal is less straightforward compared to the specular reflectrometry. For the specular reflectometry it is sufficient to normalize the intensity to the total reflection intensity. The diffuse scattering intensity, on the other hand, varies with α, $\Delta 2\theta$ and $\Delta\beta$ through $\frac{|t(\alpha)|^2}{\sin\alpha}\Delta 2\theta\Delta\beta$, in addition to the primary beam intensity I_0 (Equation 6 and Equation 9). An accurate α alignment and a correct calculation of $\Delta 2\theta$ and $\Delta\beta$ are necessary to provide the correct value of $\frac{|t(\alpha)|^2}{\sin\alpha}\Delta 2\theta\Delta\beta$, such that normalisation to I_0 can directly yield R_{pseudo} (Figure 4 and Figure 5). Practically, $\frac{|t(\alpha)|^2}{\sin\alpha}\Delta 2\theta\Delta\beta$ is held constant when the configuration of a fixed incidence instrument is not changed. Hence it can also be measured from a reference sample, e.g. water, to be applied to the other measurements from the same instrument.

S10. Q_{xy} along the GIXOS profile

GIXOS data (intensity versus β) is typically taken at one 2θ. Q_{xy} along the GIXOS profile hence increases at larger Q_z. Figure S8 shows the variation of Q_{xy} along the GIXOS profiles at five off-specular positions including the one in the plane of incidence ($2\theta = 0$, blue). At every 2θ, Q_{xy} is larger at higher Q_z. The largest deviation of Q_{xy} along Q_z is found in the plane of incidence. Q_{xy} increases from 0 to about 0.06 Å^{-1} when Q_z increases from 0 to 1 Å^{-1}. This (blue line, Figure S8) is the lower boundary of the accessible Q_{xy} under the fixed grazing incidence condition. This is the same as the Q-space mapping used in the grazing incidence geometry (Baker et al., 2010) to calculate the $\boldsymbol{Q}$-vectors from the scattering angles. This mapping is often referred to as the “Ewald correction”.

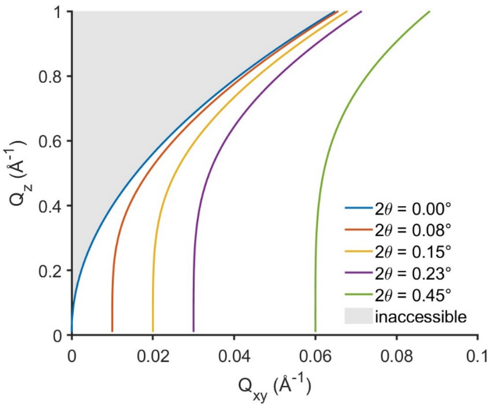

Figure S8 Q_{xy} along the GIXOS profile at several off-specular positions 2θ. Grey shading region is inaccessible under the fixed grazing incidence condition. Here the incident energy and angle are set to 15 keV and 0.07°. The chosen 2θ-s correspond to $Q_{xy}\big|_{\beta=0}$ of 0, 0.01, 0.02, 0.03 and 0.06 Å^{-1}, used in the study for the simple liquid samples.

S11. Q_{xy}-dependency of the diffuse scattering R* of the CTAB Gibbs adsorption layer

The diffuse scattering R^* of the Gibbs adsorption layer of CTAB from its 0.6 mM solution in water follows the $Q_{xy}^{\eta-2}$ dependency of CWM. Hence Equation 8-9 are applicable for this layer to derive its pseudo reflectivity. This dependency is due to the close-to-zero bending modulus of the layer.

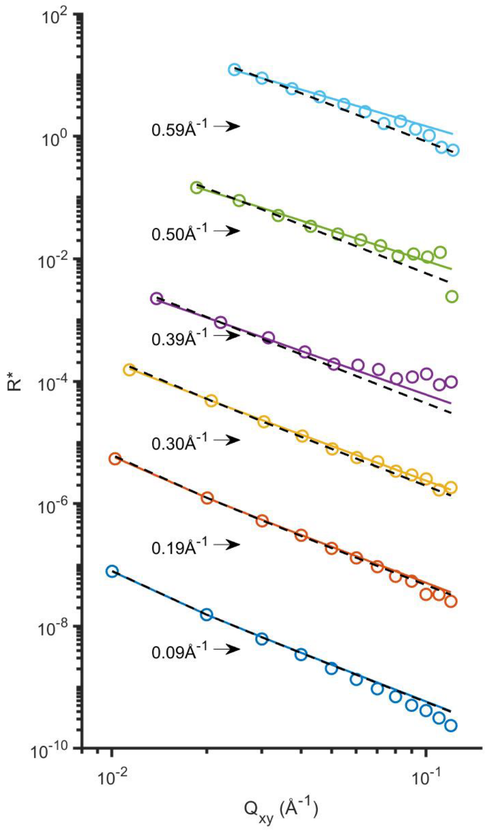

Figure S9 Q_{xy}-dependency of R^* of the CTAB Gibbs adsorption layer from a 0.6 mM solution in water from the P08 measurement. Data (circles) at different Q_z are color-coded and offset by a factor of 10 from each other, while the corresponding Q_z-values are given on the left. Solid lines are the theoretical $Q_{xy}^{\eta-2}$ drop given by γ = 45 mN/m and T = 292K at each Q_z according to Equation 6, and are normalised to R^* at the lowest Q_{xy}. The black dashed lines show simulated curves with Q_{xy}^{-2} dependency (η =0) for referencing.

References

Ashiotis, G., Deschildre, A., Nawaz, Z., Wright, J. P., Karkoulis, D., Picca, F. E. & Kieffer, J. (2015). *J Appl Crystallogr* **48**, 510-519.

Baker, J. L., Jimison, L. H., Mannsfeld, S., Volkman, S., Yin, S., Subramanian, V., Salleo, A., Alivisatos, A. P. & Toney, M. F. (2010). *Langmuir* **26**, 9146-9151.

Braslau, A., Pershan, P. S., Swislow, G., Ocko, B. M. & Als-Nielsen, J. (1988). *Phys. Rev. A* **38**, 2457-2470.

Dai, Y., Lin, B., Meron, M., Kim, K., Leahy, B. & Shpyrko, O. G. (2011). *Journal of Applied Physics* **110**, 102213.

Feidenhans'l, R. (1989). *Surf. Sci. Rep.* **10**, 105-188.

Fradin, C., Braslau, A., Luzet, D., Smilgies, D., Alba, M., Boudet, N., Mecke, K. & Daillant, J. (2000). *Nature* **403**, 871-874.

Fukuto, M., Heilmann, R. K., Pershan, P. S., Griffiths, J. A., Yu, S. M. & Tirrell, D. A. (1998). *Phys. Rev. Lett.* **81**, 3455-3458.

Hura, G., Sorenson, J. M., Glaeser, R. M. & Head-Gordon, T. (2000). *J. Chem. Phys.* **113**, 9140-9148.

Mechler, S., Pershan, P. S., Yahel, E., Stoltz, S. E., Shpyrko, O. G., Lin, B., Meron, M. & Sellner, S. (2010). *Phys. Rev. Lett.* **105**, 186101.

Murphy, B. M., Greve, M., Runge, B., Koops, C. T., Elsen, A., Stettner, J., Seeck, O. H. & Magnussen, O. M. (2014). *Journal of synchrotron radiation* **21**, 45-56.

Oliveira, R. G., Schneck, E., Quinn, B. E., Konovalov, O. V., Brandenburg, K., Gutsmann, T., Gill, T., Hanna, C. B., Pink, D. A. & Tanaka, M. (2010). *Physical review. E, Statistical, nonlinear, and soft matter physics* **81**, 041901.

Orthaber, D., Bergmann, A. & Glatter, O. (2000). *J Appl Crystallogr* **33**, 218-225.

Pershan, P. S. (2000). *Colloids and Surfaces A: Physicochemical and Engineering Aspects* **171**, 149-157.

Schwartz, D. K., Schlossman, M. L., Kawamoto, E. H., Kellogg, G. J., Pershan, P. S. & Ocko, B. M. (1990). *Phys. Rev. A* **41**, 5687-5690.

Shen, C., Kirchhof, R. & Bertram, F. (2022). *Journal of Physics: Conference Series* **2380**, 012047.

Shpyrko, O., Fukuto, M., Pershan, P., Ocko, B., Kuzmenko, I., Gog, T. & Deutsch, M. (2004). *Phys. Rev. B* **69**, 245423.

Shpyrko, O., Huber, P., Grigoriev, A., Pershan, P., Ocko, B., Tostmann, H. & Deutsch, M. (2003). *Phys. Rev. B* **67**, 115405.

Sloutskin, E., Tamam, L., Sapir, Z., Ocko, B. M., Bain, C. D., Kuzmenko, I., Gog, T. & Deutsch, M. (2022). *Langmuir* **38**, 12356-12366.

Tostmann, H., DiMasi, E., Pershan, P. S., Ocko, B. M., Shpyrko, O. G. & Deutsch, M. (1999). *Phys. Rev. B* **59**, 783-791.

Tristram-Nagle, S. & Nagle, J. F. Private communication.
